# The Prebiotic and Techno‐Functional Potential of Microbial Exopolysaccharides for Human Health and Food Systems

**DOI:** 10.1111/1541-4337.70406

**Published:** 2026-01-31

**Authors:** Md. Abdur Razzak, Hye Kim, Md. Ariful Haque, Min Ji Jang, Sehyeon Song, Anishka Talari, Lakshmi Devi Chittepu, Seockmo Ku

**Affiliations:** ^1^ Department of Food Science and Technology Texas A&M University College Station Texas USA

**Keywords:** exopolysaccharide, food application, gut microbiota, prebiotic, systemic health, techno‐functional

## Abstract

Microbial exopolysaccharides (EPS) represent a diverse class of biopolymers holding considerable promise as functional food ingredients. This review analyzes the dual function of microbial EPS as a candidate for prebiotic agents and techno‐functional additives. Differentiating between broad chemical categories and validated functional ingredients is critical. Many EPS are resistant to digestion; however, only those that meet the criteria established by the International Scientific Association for Probiotics and Prebiotics (ISAPP)—notably, selective utilization by host microorganisms that confer health benefits—are classified as prebiotics. This review examines the “EPS‐microbiota‐host axis,” demonstrating how specific prebiotic EPS influence gut ecology to produce short‐chain fatty acids (SCFAs). These metabolites regulate immunomodulatory and systemic metabolic homeostasis, which may have therapeutic benefits for conditions including diabetes and obesity. It is essential to differentiate microbiota‐mediated effects from direct biological mechanisms associated with “bioactive EPS,” including immune receptor modulation, which operate independently of fermentation and should not be equated with postbiotics. Beyond health, the structural complexity underpinning these activities confers significant techno‐functional utility. We explore EPS as natural emulsifiers, rheology modifiers, and biodegradable packaging materials, emphasizing their stabilizing roles in dairy and bakery systems. Finally, we examine techno‐economic barriers, particularly production scalability, that hinder widespread adoption and discuss the potential of synthetic biology to engineer “designer” polysaccharides. This synthesis clarifies the precise roles of microbial EPS as sustainable, multifunctional ingredients in developing next‐generation healthy foods.

## 1 Introduction

The human gastrointestinal tract harbors a diverse community of microorganisms known as the gut microbiota, which significantly impacts host health besides its functions in digestion and nutrient absorption (C. Zhang, Liu, et al. 2023). This symbiotic relationship has generated interest in the modulation of gut microbiota to enhance well‐being, particularly using prebiotics (Bindels et al. 2015). The International Scientific Association for Probiotics and Prebiotics (ISAPP) defines prebiotics as “a substrate that is selectively utilized by host microorganisms, conferring a health benefit” (Hutkins et al. 2024; Marco et al. 2021). It is important to note that this definition is function‐driven; a compound is not classified as a prebiotic solely based on indigestibility or microbial origin. To qualify, an ingredient must demonstrate digestive resistance, quantifiable modulation of the microbiome, a causal link between this modulation and a health outcome, and proven safety (Hutkins et al. 2024).

Historically, prebiotics were defined as nondigestible carbohydrates that promote the growth of *Bifidobacterium* species, exhibiting a “bifidogenic effect.” Examples include fructo‐oligosaccharides (FOS), inulin, and galacto‐oligosaccharides (GOS) (S. Fan et al. 2024). However, culture‐independent sequencing techniques, including 16S rRNA gene sequencing and shotgun metagenomics, have revealed the significant complexity of the gut microbiota. These studies indicate that *Bifidobacteria* constitute only a minor component of adult microbiota (Wensel et al. 2022). Essential butyrate‐producing Clostridia, such as *Faecalibacterium*, *Roseburia*, and *Eubacterium*, are now recognized as critical for colonic health and immune regulation (Singh et al. 2023). Furthermore, the understanding of microbial metabolism has expanded to include ecological interactions such as cross‐feeding, where primary fermenters produce metabolites (e.g., acetate and lactate) that serve as substrates for secondary degraders (Fu et al. 2019). This ecological perspective has broadened the scope of prebiotic candidates beyond simple oligosaccharides to more complex biopolymers that can sustain diverse microbial networks. However, determining which of these biopolymers satisfy the rigorous selectivity criteria, however, remains a primary scientific challenge.

**FIGURE 1:**
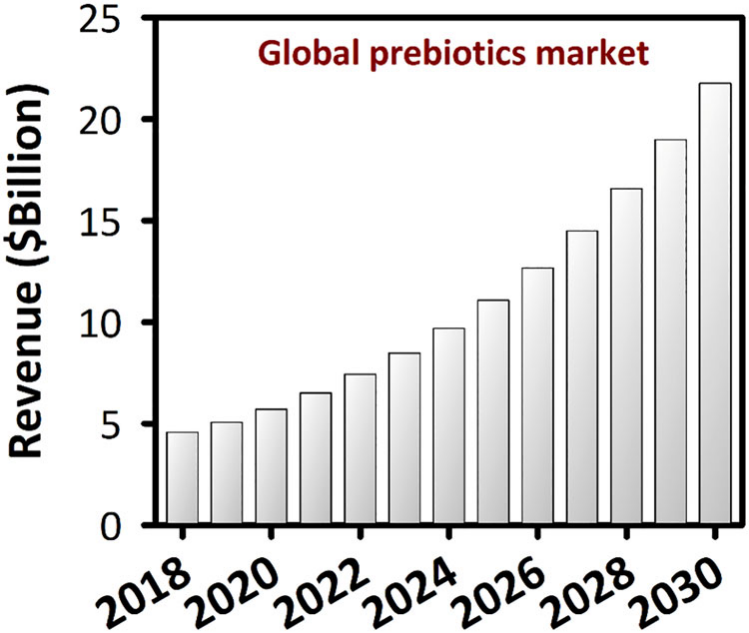
The global prebiotics market exhibits significant growth, with projected revenue anticipated to rise from 2018 to 2030 (Grand View Research 2025).

The commercial significance of these functional ingredients is substantial. The global prebiotics market is projected to expand from $8.51 billion in 2023 to $21.77 billion by 2030, driven by a compound annual growth rate of 14.3% (Figure 1) (Grand View Research 2025). Although the current market is dominated by established plant‐derived fibers like inulin and FOS, there is a growing industrial and scientific interest in alternative sources that offer superior functionality.

In this context, microbial exopolysaccharides (EPS)—biopolymers secreted by bacteria, fungi, and algae—have emerged as a frontier in prebiotic research. It is crucial to distinguish between EPS as a broad chemical category and validated prebiotics. To meet the rigorous ISAPP criteria, a specific EPS structure must exhibit digestive resistance, selective utilization by beneficial microorganisms, and a resultant health benefit. Numerous EPS may exhibit non‐fermentable properties or functions through direct signaling mechanisms; therefore, they ought to be categorized as bioactive biopolymers instead of prebiotics. This review maintains these distinctions to prevent the confusion frequently encountered in the literature. Unlike plant‐derived fibers, microbial EPS offer unique structural complexity and biodiversity that remain largely untapped. Recent reviews have systematically documented EPS chemical structures or focused on specific health benefits. However, the literature often remains fragmented, addressing technological applications independently from biological activity in the gut (Balthazar et al. 2022; Kaur and Dey 2023; Korcz and Varga 2021; J. Kumari et al. 2025; Pourjafar et al. 2023). Thus, there is a notable scarcity of comprehensive syntheses that bridge the gap between food science and clinical nutrition.

This review addresses this critical gap by integrating the dual function of microbial EPS as both high‐performance techno‐functional additives and potent prebiotic agents. To facilitate a clear understanding of this framework, the article is organized into sequential sections. We begin by summarizing the sources and biosynthesis pathways of EPS to provide the necessary structural context. We then establish the mechanistic framework of the “EPS‐microbiota‐host axis,” detailing how specific structural features dictate digestive resistance and drive selective fermentation. Building on this, we critically analyze the evidence for systemic health benefits, including metabolic, immunological, and antimicrobial homeostasis. Subsequently, we examine the techno‐functional attributes of these biopolymers—such as emulsification, gelation, and encapsulation—and their practical integration into food systems. Finally, the review discusses existing techno‐economic barriers and regulatory landscapes, concluding with future perspectives on engineering “designer” polysaccharides for next‐generation healthy foods. A summary of the review methodology is provided in Box 1.

**BOX 1 | Review methods**

**Search strategy**: This narrative review synthesizes literature from the past two decades, with a specific emphasis on recent advancements from 2020 through mid‐2025. Bibliographic databases utilized included PubMed, Web of Science, and Google Scholar.

**Key search terms**: Queries encompassed combinations of terms such as “microbial exopolysaccharide,” “prebiotic,” “gut microbiota,” “SCFA,” “techno‐functional properties,” “food application,” and specific bioactive outcomes (e.g., anti‐obesity EPS, immunomodulation EPS).

**Screening criteria**: The selection process prioritized original research, systematic reviews, and meta‐analyses that

- Provided clear structural characterization (e.g., composition and molecular weight) of the EPS.
- Examined fundamental biological mechanisms, such as selective fermentation, enzyme inhibition, or signaling pathways.
- Presented distinct techno‐functional applications or specific health‐related benefits.
- Addressed scalable production techniques or integration into food products.

## 2 Microbial EPS: Their Sources, Classification, and Biosynthesis

### 2.1 Sources of EPS

Microorganisms that produce EPS are widespread, occupying various ecological niches worldwide. The producers include prokaryotes (eubacteria and archaea) and eukaryotes (fungi, phytoplankton, and algae), demonstrating the extensive ability for EPS biosynthesis among microorganisms (Donot et al. 2012).

#### 2.1.1 Bacteria

Bacteria represent a significant and industrially relevant source of EPS, with notable instances observed across various genera (Kaur and Dey 2023). Xanthan gum, derived from *Xanthomonas campestris*, is among the most commercially significant biopolymers. Additional significant industrial gums are gellan gum, synthesized by *Sphingomonas paucimobilis*, and alginate, produced by *Azotobacter vinelandii* as well as the opportunistic pathogen *Pseudomonas aeruginosa*. Species, such as *Acetobacter xylinum*, are recognized for their ability to produce a pure form of bacterial cellulose (Kaur and Dey 2023).

Lactic acid bacteria (LAB) are also major producers of EPS. *Leuconostoc mesenteroides* synthesizes dextran, whereas species, such as *Lactobacillus helveticus* and *Lactobacillus rhamnosus*, generate various unique heteropolysaccharides (HePSs) essential for the texture of fermented dairy products (Kaur and Dey 2023; Korcz and Varga 2021). In the medical field, high‐value EPS, such as hyaluronic acid, can be synthesized by bacteria, including *Streptococcus zooepidemicus* and *Bacillus subtilis*. Moreover, several members of the Enterobacteriaceae family, such as *Escherichia coli*, *Shigella* spp., *Salmonella* spp., and *Enterobacter* spp., are recognized for their production of capsular polysaccharides (CPS), with colanic acid from *E. coli* being a well‐studied example (Kaur and Dey 2023; Korcz and Varga 2021).

#### 2.1.2 Fungi

Numerous fungi are acknowledged as significant sources of diverse EPS, with various genera yielding specific types of glucans (W. Wang et al. 2022). *Aureobasidium pullulans* represents an important commercial example due to its ability to produce pullulan, a linear α‐glucan utilized in the manufacture of edible films (Paul et al. 2023). Various fungi synthesize specific polysaccharides; for example, the medicinal mushroom *Ganoderma* is recognized for ganoderan, a β‐glucan exhibiting immunomodulatory properties, whereas *Sclerotium rolfsii* generates the gelling agent scleroglucan, and *Schizophyllum* commune produces schizophyllan (Paul et al. 2023; W. Wang et al. 2022). Common fungi such as *Aspergillus* and *Penicillium* synthesize various EPS, including distinct β‐glucans. Similarly, diverse genera, including *Tuber*, *Colletotrichum*, *Fusarium*, and *Monascus*, are recognized for producing β‐glucans as their primary EPS. These polysaccharides share a fundamental backbone structure, although specific branching varies among species (Donot et al. 2012; Rana and Upadhyay 2020; W. Wang et al. 2022).

#### 2.1.3 Algae

Microalgae are also increasingly acknowledged for their capacity to synthesize EPS. EPS are generated by several microalgal phyla, including Charophyta, Chlorophyta, Ochrophyta, Miozoa, Bacillariophyta, Haptophyta, and Rhodophyta (He et al. 2025). Prominent EPS‐producing species are *Dunaliella tertiolecta*, characterized by an EPS composition mainly of glucose, galactose, mannose, and rhamnose, and *Scenedesmus acuminatus*, which produces an EPS abundant in rhamnose, mannose, galactose, glucose, and xylose (He et al. 2025; Paul et al. 2023). Furthermore, red algae, including *Porphyridium sordidum*, *Porphyridium cruentum*, and *Rhodella* sp., are recognized for their production of sulfated EPS. Their EPS primarily comprise galactose and xylose units, which are associated with sulfate (SO_4_^2−^) groups (Donot et al. 2012; He et al. 2025; Paul et al. 2023).

### 2.2 Classification of EPS Based on Chemical Composition

**FIGURE 2:**
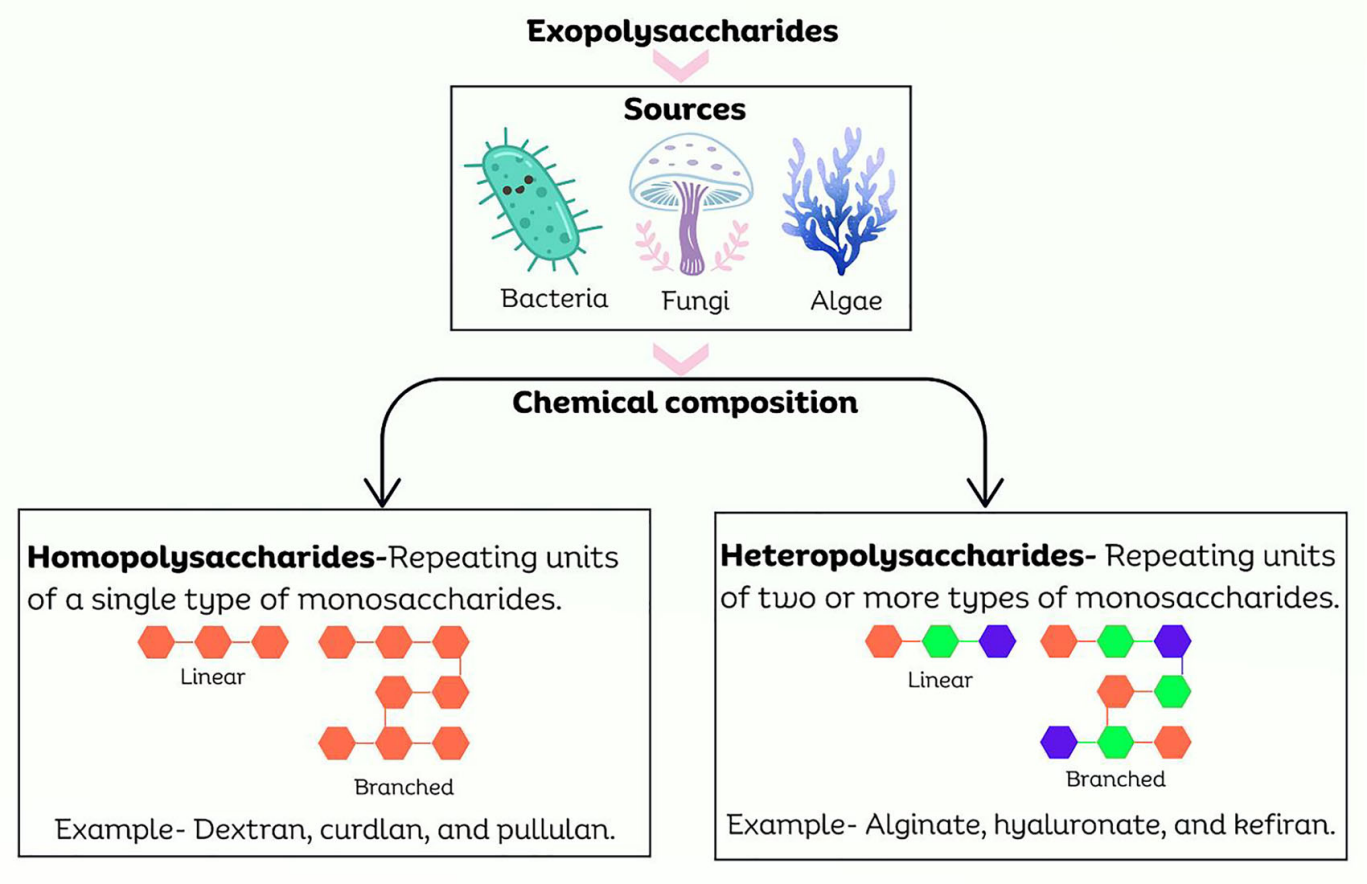
Sources and categorization of exopolysaccharides based on their chemical structure. The figure was created in Canva.com.

Microbial EPS exhibits considerable structural diversity, which directly affects their functional properties. These biopolymers are primarily classified into two main categories: homopolysaccharides (HoPSs) and HePSs based on the composition of their constituent monosaccharide units (Lynch et al. 2018a). This classification establishes an essential framework for comprehending their structural complexity and potential functionalities. Table 1 and Figure 2 present the characteristics of microbial HoPS and HePS.

**TABLE 1:** Classification and representative examples of microbial exopolysaccharides.

Type of EPS	Example of EPS	Primary monomer(s)	Key microbial producer(s)	Key structural feature	References
Homopolysaccharide (HoPS)	Dextran	Glucose	*Leuconostoc mesenteriodes*, *Streptococcus mutans*	Predominantly α‐1,6 glucan with various branches	J. Wu, Han, et al. (2023)
	Curdlan	Glucose	*Agrobacterium* sp.	Linear β‐1,3‐glucan	Kaur and Dey (2023)
	Bacterial cellulose	Glucose	*Acetobacter* spp., *Rhizobium* sp.	Linear β‐1,4‐glucan	Kaur and Dey (2023)
	Pullulan	Glucose (as maltotriose)	*Aureobasidium pullulans*	Maltotriose units linked by α‐1,6 bonds, internal α‐1,4 bonds	Paul et al. (2023)
Heteropolysaccharide (HePS)	Xanthan	Glucose, mannose, glucuronic acid	*Xanthomonas campestris*	Glucose backbone with trisaccharide side chains	Paul et al. (2023)
	Alginate	Mannuronic acid, guluronic acid	*Azotobacter vinelandii*, *Pseudomonas aeruginosa*	Linear copolymer of M and G blocks	Rana and Upadhyay (2020)
	Gellan	Glucose, glucuronic acid, rhamnose	*Sphingomonas paucimobilis*	Linear tetrasaccharide repeating unit	Sun and Zhang (2021)
	Hyaluronic acid	Glucuronic acid, *N*‐acetyl‐glucosamine	*Streptococcus equisimilis/zooepidemicus*, *Bacillus subtilis*	Repeating disaccharide units	J. Kumari et al. (2025)
	Kefiran	Glucose and galactose	*Lactobacillus kefiranofaciens*	Complex, often branched, with a specific sugar composition of glucose and galactose	Lynch et al. (2018b)

Abbreviation: EPS, exopolysaccharides.

#### 2.2.1 Homopolysaccharides

HoPSs are defined by their simple structure, comprising repeating units of a single monosaccharide, typically glucose or fructose (Lynch et al. 2018b). Despite their monomeric simplicity, HoPSs display various structural architectures, including both unbranched (linear) and highly branched forms. Notable examples include glucans, polymers exclusively made of glucose units, and fructans, polymers composed of fructose. Examples of specific glucans include dextran, a bacterial HoPS primarily synthesized by *L. mesenteroides* and *Streptococcus mutans*, which is characterized by α‐1,6 glycosidic bonds, as well as α‐1,2, α‐1,3, and α‐1,4 linkages (Rana and Upadhyay 2020).

Curdlan, a glucose‐only HoPS synthesized by *Agrobacterium* sp., is characterized by its β‐1,3‐linked glucose units (J. Kumari et al. 2025). Bacterial cellulose, a glucose polymer, is produced by *Acetobacter* spp. and *Rhizobium* sp. Pullulan is a unique glucan synthesized by the fungus *A. pullulans*, consisting of repeating maltotriose units. Levan and inulin are also common examples of fructans classified as HoPS (J. Kumari et al. 2025; Rana and Upadhyay 2020).

#### 2.2.2 Heteropolysaccharides

HePSs are characterized by their complex structures, consisting of repeating units of two or more distinct monosaccharides, generally comprising three to seven different monomer units (Lynch et al. 2018b). These polymers frequently display complex, branched structures and can include a wide range of monosaccharides, such as glucose, fructose, galactose, mannose, rhamnose, fucose, arabinose, xylose, *N*‐acetylglucosamine, and various uronic acids. They may also include noncarbohydrate groups, such as phosphate or acetyl groups.

Examples of HePS include kefiran, which is produced by *Lactobacillus kefiranofaciens* and comprises glucose, mannose, and glucuronic acid (Lynch et al. 2018b). Alginate, produced by bacteria including *A. vinelandii* and *P. aeruginosa*, consists of mannuronic acid and guluronic acid (J. Kumari et al. 2025; Tiwari et al. 2024). Gellan, synthesized by *S. paucimobilis*, comprises glucose, glucuronic acid, and rhamnose as its components. Hyaluronic acid, produced by *Streptococcus equisimilis/S. zooepidemicus* and *B. subtilis*, consists of repeating disaccharide units of glucuronic acid and *N*‐acetylglucosamine (J. Kumari et al. 2025).

A key distinction in microbial EPS is observed between HoPS, generally neutral in charge, and HePS, most of which are polyanionic (J. Kumari et al. 2025). The fundamental chemical distinction, along with significant variations in monomer compositions, linkage types, and other substituents, highlights the considerable structural variability present in these biopolymers (Lynch et al. 2018b). This structural blueprint determines the distinct physicochemical properties of each polymer, such as viscosity, gelation characteristics, and emulsifying abilities (Rana and Upadhyay 2020). The structure–function relationship has important implications for industrial applications, allowing for precise control over the final properties of the biopolymer. The selection of specific microbial strains or the application of advanced genetic engineering techniques enables the customization of EPS characteristics to fulfill precise functional requirements. This advancement signifies a fundamental change from merely discovering natural polymers to intentionally designing them via targeted microbial biosynthesis, enabling enhanced control over material performance (Donot et al. 2012; Kaur and Dey 2023; J. Wu, Han, et al. 2023).

### 2.3 Biosynthesis of Microbial EPS

**FIGURE 3:**
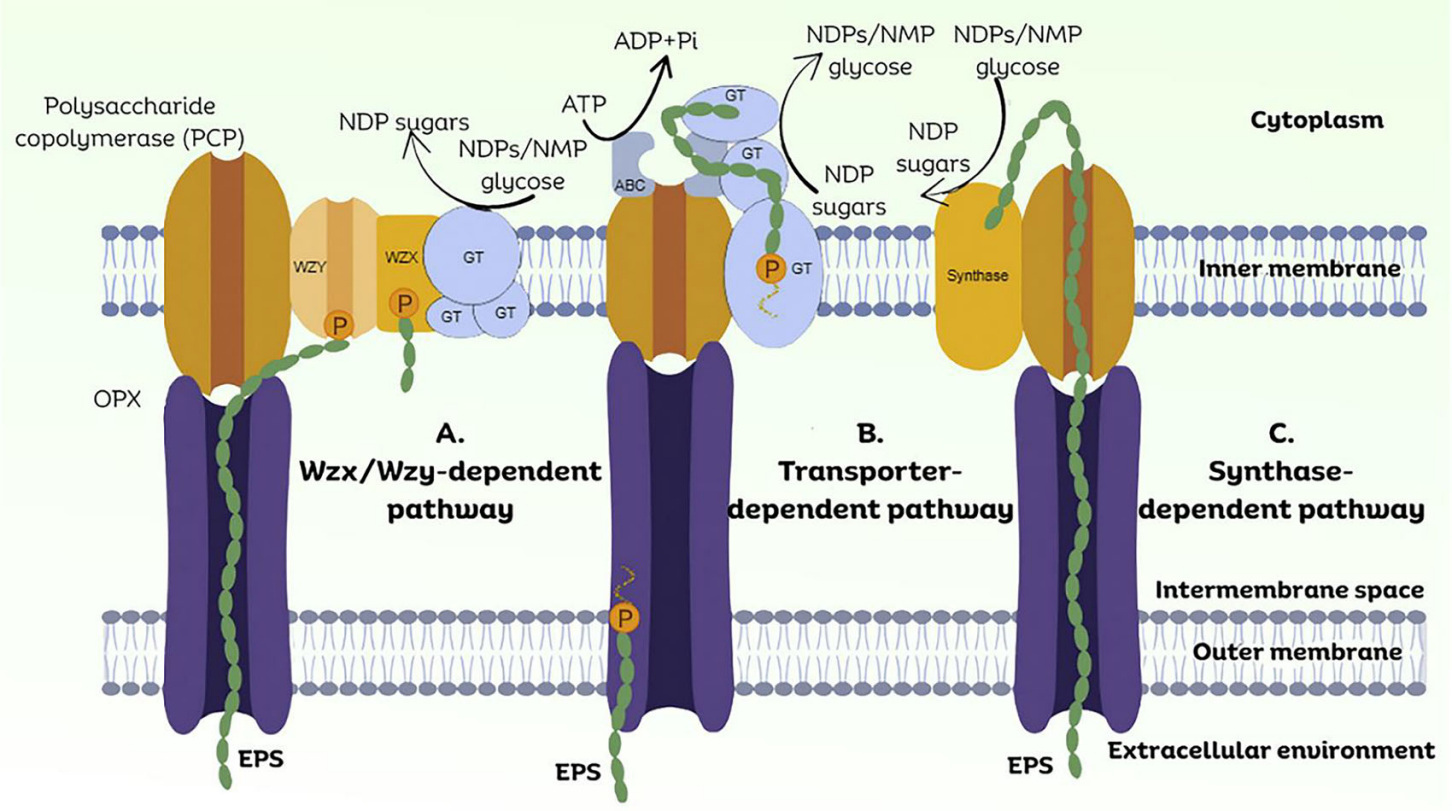
Overview of three distinct exopolysaccharide synthesis pathways. NDP refers to nucleoside diphosphate, NMP denotes nucleoside monophosphate, and GT stands for glycosyltransferase. EPS, exopolysaccharides. *Source*: Figure adapted from Laubach et al. (2021) with permission from Elsevier. The image was modified to enhance visual clarity using Canva.com.

The biosynthesis of microbial EPS is a complex process that encompasses various biochemical pathways and regulatory mechanisms, which differ according to the specific polymer and organism involved. The production of EPS is closely associated with the growth phase of microorganisms and is significantly influenced by environmental factors. For most EPS, it takes place intracellularly prior to the transport of the completed macromolecule to the extracellular space. At present, four primary pathways for EPS production are identified. Three of these pathways—the Wzx/Wzy‐dependent, the adenosine triphosphate (ATP)‐binding cassette (ABC) transporter‐dependent, and the synthase‐dependent—depend on intracellular synthesis, whereas a fourth mechanism entails direct extracellular synthesis (Figure 3) (J. Kumari et al. 2025; Zang et al. 2025). This review addresses the overall topic and functions of the different pathways. Readers interested in detailed explanations and specific pathways for bacteria, fungi, and algae are encouraged to read the following articles (S. S. Ali et al. 2025; Chaudhuri et al. 2025; Stephens et al. 2023; J. Wu, Han, et al. 2023).

#### 2.3.1 Wzx/Wzy‐Dependent Pathway

This pathway, frequently found in Gram‐negative bacteria like *E. coli*, facilitates the synthesis of HePS (J. Kumari et al. 2025). The process begins in the cytoplasm, utilizing nucleotide‐activated sugars, such as UDP‐glucose, to construct an oligosaccharide repeating unit. The assembly process is catalyzed by glycosyltransferases (GTs) acting on an undecaprenol diphosphate lipid carrier in the inner membrane. Subsequently, the flippase protein Wzx facilitates the transport of the completed repeating unit across the cytoplasmic membrane into the periplasm. Within the periplasm, the Wzy protein facilitates the polymerization of these units into a high‐molecular‐weight (Mw) polysaccharide. The final export of the polymer to the cell surface is mediated by proteins from the polysaccharide co‐polymerase (PCP) and outer membrane polysaccharide export (OPX) families (J. Kumari et al. 2025; J. Wu, Han, et al. 2023).

#### 2.3.2 ABC Transporter‐Dependent Pathway

This pathway is also common in Gram‐negative bacteria and is primarily linked to the biosynthesis of CPS (Laubach et al. 2021). Polymerization occurs on the cytoplasmic side of the inner membrane, facilitated by GTs. The synthesis of HePS requires multiple GTs, whereas HoPS may utilize a single GT‐containing operon. A notable characteristic of this pathway is the presence of a conserved glycolipid at the reducing terminus of the polymer. The translocation of the completed polysaccharide across the inner membrane to the cell surface is facilitated by a tripartite efflux‐like complex. This complex includes an ABC transporter located on the inner membrane, in addition to PCP and OPX proteins that traverse the periplasmic space (J. Kumari et al. 2025; Laubach et al. 2021).

#### 2.3.3 Synthase‐Dependent Pathway

The synthase‐dependent pathway employs a singular synthase protein, typically functioning as a subunit in an envelope‐spanning multi‐protein complex, to facilitate both polymerization and translocation (Rana and Upadhyay 2020). This mechanism is characterized by its ability to synthesize and secrete complete polymer strands across cell membranes and walls without requiring a Wzx flippase for the translocation of repeating units. This pathway is exemplified by the synthesis of hyaluronan (HA) in bacteria like *S. equisimilis*. This process synthesizes an HoPS from two different nucleotide‐sugar precursors: UDP‐glucuronic acid and UDP‐*N*‐acetylglucosamine. A membrane‐embedded enzyme, HA synthase (HAS), concurrently polymerizes the elongating polysaccharide chain and translocates it across the cytoplasmic membrane. This pathway is characterized by the absence of a lipid acceptor molecule throughout the entire process (Laubach et al. 2021; Rana and Upadhyay 2020).

#### 2.3.4 Extracellular Synthesis (Sucrase‐Mediated) Pathway

This pathway is quite distinct, as it takes place entirely in an extracellular environment, independent of central cellular carbon metabolism (J. Kumari et al. 2025). It is utilized in the synthesis of certain HoPS, including glucans (e.g., dextran and mutan) and fructans (e.g., levan). The process depends on the secretion of specific enzymes, such as glycan‐sucrases, which target extracellular substrates, generally disaccharides. These enzymes facilitate the hydrolysis of the substrate and harness the released energy to transfer monosaccharide residues to an elongating polysaccharide chain. This mechanism produces polymers with restricted structural variation and relies solely on the availability of external substrates and the activity of the secreted sucrase (J. Kumari et al. 2025; Rana and Upadhyay 2020).

The yield and structural quality of synthesized EPS are significantly affected by culturing conditions, as well as the genetic characteristics of microbial species and strains. The composition of the nutritional medium is crucial; for instance, the carbon‐to‐nitrogen ratio is a significant factor, as an excess of carbon in nitrogen‐limiting conditions can redirect the cell's metabolism from growth to increased EPS production (Kaur and Dey 2023). The inclusion of cost‐effective substrates, along with optimal concentrations of phosphates, salts, and essential metal ions that serve as enzymatic cofactors, is essential for supporting biosynthetic pathways. Additionally, physical parameters require precise control. The pH and temperature of the culture directly affect enzyme kinetics, whereas agitation and aeration rates facilitate uniform nutrient distribution and satisfy the oxygen requirements for cellular respiration. The intensity, duration (photoperiod), and spectral quality of light directly regulate the energy supply for synthesis in phototrophic microorganisms such as cyanobacteria. For an in‐depth review of these parameters and their impact on EPS production, readers are advised to read these articles addressing these topics (J. Kumari et al. 2025; L. Liu, Zhang, et al. 2025; J. Wu, Han, et al. 2023).

## 3 The Prebiotic Potential of Microbial EPS: Mechanisms of Action

**FIGURE 4:**
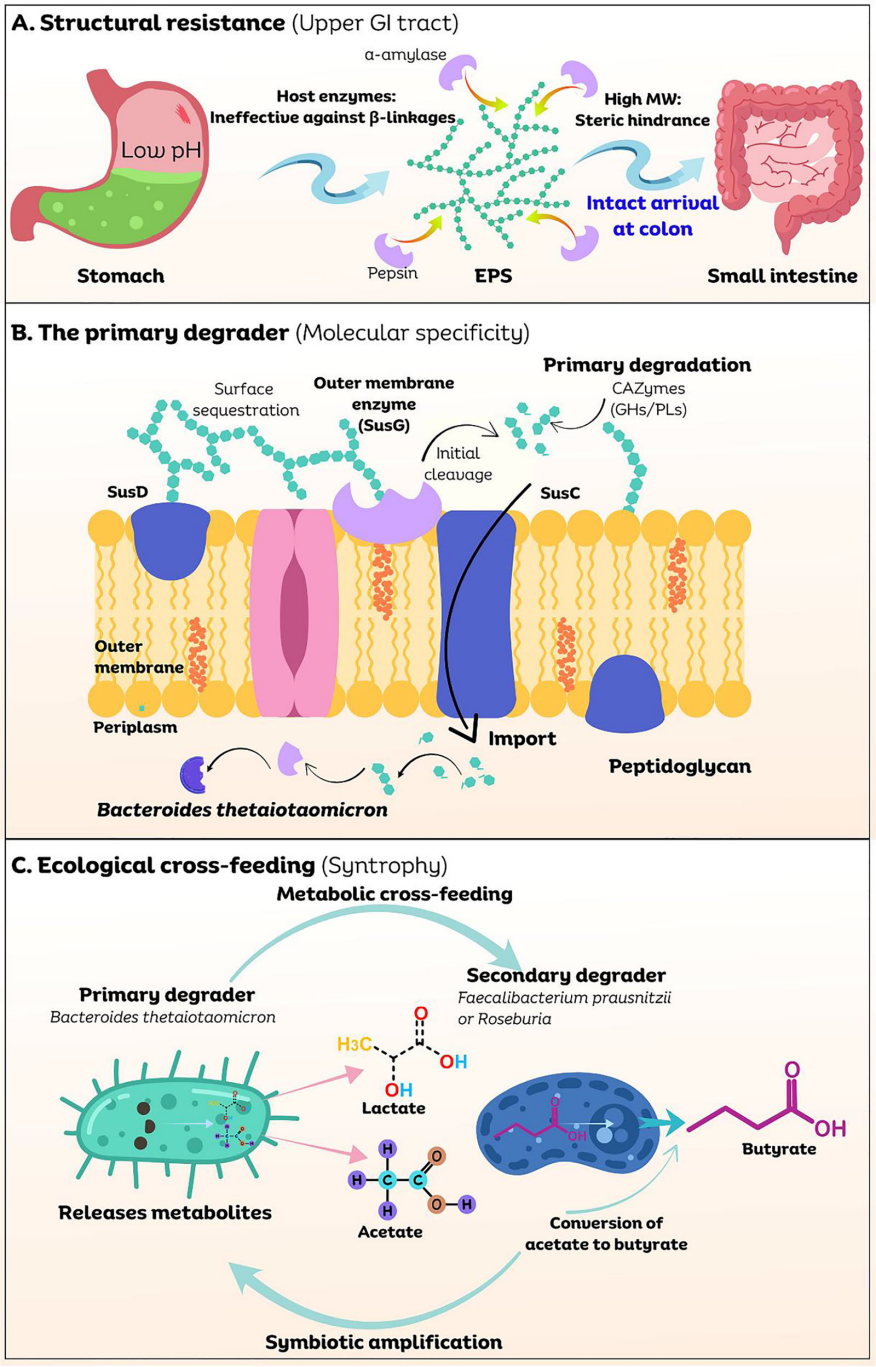
The mechanistic cascade of EPS prebiotic activity. (A) Resistance to host digestion: High molecular weight and specific β‐glycosidic linkages prevent upper GI hydrolysis, ensuring the polymer reaches the colon intact. (B) Molecular specificity: Primary degraders (e.g., *Bacteroides*) utilize polysaccharide utilization loci (PULs) to sequester EPS. SusD‐like proteins bind the polymer, whereas SusG‐like enzymes and SusC‐like transporters facilitate cleavage and import. (C) Ecological cross‐feeding: Primary degraders release intermediates (acetate and lactate) that support secondary fermenters (e.g., *Faecalibacterium* and *Roseburia*). These species utilize the butyryl‐CoA:acetate CoA‐transferase pathway to produce butyrate, enhancing host metabolic benefits. EPS, exopolysaccharides. Source: Figure was created in Canva.com.

The potential prebiotic mechanism of candidate EPS can be delineated in the subsequent steps (Figure 4) (Bedu‐Ferrari et al. 2022; S. Fan et al. 2023; Payling et al. 2020). The structural complexity by itself does not ensure prebiotic function. It is essential to differentiate between EPS that exhibit confirmed clinical benefits (confirmed prebiotics), those that possess theoretical fermentability (candidate prebiotics), and those that function through direct interaction with the host (bioactive biopolymers). Thus, this review classifies EPS into three distinct categories based on the strength of the available biological evidence, highlighting the varying levels of substantiation among them (Box 2).

**BOX 2 | Evidence hierarchy for EPS prebiotic potential**

- **Category A: Confirmed prebiotics (clinical evidence)**. EPS in this category have shown specific digestive resistance and selective fermentation in human clinical trials. Evidence comprises alterations in the composition of the human gut microbiota, the synthesis of short‐chain fatty acids (SCFAs), and observable health benefits.
- **Category B: Candidate prebiotics (in vitro and/or in vivo models)**. EPS demonstrates mechanistic plausibility; however, it lacks confirmation in human studies. Evidence is obtained from in vitro fecal fermentation models or in vivo animal studies that elucidate specific mechanisms (CAZymes/PULs).
- **Category C: Bioactive or techno‐functional EPS (non‐prebiotic)**. EPS are characterized by techno‐functional properties and structural features that indicate digestive resistance; however, there is an absence of direct evidence for selective fermentation.

For validated candidates (Categories A and B in Box 2), the mechanism generally functions as follows: First, prebiotics are resistant to host digestion, enabling their arrival in the colon in an intact form. Second, upon arrival, they are subjected to selective fermentation by beneficial gut microbes equipped with the necessary enzymatic tools for their breakdown. Third, this process regulates microbiota by enhancing the proliferation of beneficial bacteria. Fourth, this activity results in the production of bioactive metabolites, including short‐chain fatty acids (SCFAs). Finally, these metabolites are absorbed and may facilitate systemic health benefits for the host, serving as an essential communication link between the gut and the rest of the body. The following sections provide an in‐depth explanation for each step.

### 3.1 Step 1: Resistance to Host Digestion—The Structural Basis of Bioavailability

To exhibit a prebiotic effect, a compound must first successfully traverse the harsh environments of the upper gastrointestinal tract. The transit through the highly acidic stomach (pH 1.5–3.5) and the enzyme‐rich small intestine, without significant degradation or absorption, is a critical prerequisite (Figure 4A). The resistance of EPS constitutes a dynamic, multilayered defense system based in its unique and complex structural architecture, in contrast to pharmaceutical agents that frequently require artificial enteric coatings for stability (S. Fan et al. 2023; Oerlemans et al. 2021). Table 2 elaborates on the key structural features of EPS that confer resistance to digestion.

**TABLE 2:** The key structural features of exopolysaccharides (EPS) that provide resistance to digestion (Cantu‐Jungles and Hamaker 2020; Glowacki and Martens 2020; Payling et al. 2020).

Feature	Description	Resistance mechanism
Molecular weight	High (e.g., 0.5–2.0 × 10^6^ Da)	Physically hinders access and activity of digestive enzymes
Monosaccharide composition	Varied and complex, especially for heteropolysaccharides (HePS), including d‐glucose, d‐galactose, l‐rhamnose, *N*‐acetylated monosaccharides, uronic acids	Unique sugar units and sequences are not recognized or cleaved by human enzymes
Glycosidic linkages	Contains specific and often unusual linkages (e.g., β‐(1 → 3), β‐(1 → 4), α‐(1 → 2), α‐(1 → 3)) not typically targeted by human digestive enzymes	Human enzymes lack the specific hydrolase activity for these bonds
Branching degree	Highly branched and complex polymeric structures	Creates a dense, inaccessible network that sterically hinders enzyme action
Overall physical structure	Forms a protective coating around bacteria (capsule) or is released as a viscous slime	Provides a physical barrier against gastric acid, bile salts, and enzymatic attack, enhancing persistence

#### 3.1.1 Molecular Determinants of Resistance

The resistance of microbial EPS to human digestion arises from a set of distinct molecular characteristics that surpass the enzymatic capabilities of the host. Structurally, their high Mw, typically between 0.5 and 2.0 × 10^6^ Da, combined with a complex, highly branched fibrous microstructure, results in considerable steric hindrance that hinders access for digestive enzymes (Bedu‐Ferrari et al. 2022; S. Fan et al. 2023). The construction of a protective capsule or mucus layer frequently reinforces this physical barrier. Chemically, their complexity is multifaceted. EPS mostly consists of various monosaccharides, including d‐galactose and l‐rhamnose, linked by glycosidic bonds—mainly β‐(1 → 3) and β‐(1 → 4)—which are not susceptible to cleavage by human amylases that target α‐linkages in starch (Glowacki and Martens 2020). Additionally, chemical modifications involving substituent groups such as sulfate or phosphate can introduce an anionic charge, thereby modifying the polymer's conformation and hindering enzymatic recognition.

These structural properties not only prevent digestion but also influence how EPS interacts with gut bacteria during fermentation. The higher Mw, for example, results in a reduced fermentation rate by a more specialized group of bacteria (Procházková et al. 2023). This enables the EPS to remain in the gastrointestinal tract for an extended period, reaching the distal colon and potentially facilitating a prolonged release of beneficial metabolites such as SCFAs. The three‐dimensional conformation of the polymer, such as random coil, rigid rod, or helix, also significantly influences its solubility and accessibility to microbial enzymes. Furthermore, many high‐Mw EPS are recognized for their ability to enhance the viscosity of the intestinal fluid (Category C functionality) (Cantu‐Jungles and Hamaker 2020; Glowacki and Martens 2020). This effect prolongs gastrointestinal transit time, thereby extending the duration for microbial fermentation, and may also yield additional benefits, such as a reduced glycemic response. In this approach, the structural features that confer protection to the microbe simultaneously define its role as a candidate for a potent and complex prebiotic substrate.

#### 3.1.2 A Coevolutionary Advantage: Linking Microbial Protection to Host Benefit

The digestive resistance of bacterial EPS results from evolutionary pressure, providing microorganisms with essential survival mechanisms. Structurally, EPS acts as a physical shield against gastric acid and bile salts. This protection safeguards beneficial bacteria like *Lactobacillus* and *Bifidobacterium*, promoting their colonization in the human gut (Castro‐Bravo et al. 2018; Oerlemans et al. 2021). This dynamic illustrates a coevolutionary relationship in which the host's digestive system selectively promotes microbes possessing protective EPS coating. The indigestibility that facilitates microbial survival enables EPS to act as a prebiotic, arriving in the colon intact to support the resident beneficial microbial community. The alignment of microbial self‐preservation with host benefit underscores a symbiotic relationship, wherein bacterial adaptations for survival serve as mechanisms that enhance host health.

### 3.2 Step 2: Selective Fermentation—The Microbial Toolkit for Glycan Foraging

Upon arrival at the colon, the undigested EPS serves as a nutrient source for the resident microbiota. The fermentation process exhibits high selectivity, influenced by the specialized molecular mechanisms that specific bacteria have developed to decompose complex carbohydrates. Table 3 presents a summary of the enzymatic mechanisms and selectivity evidence for key EPS.

**TABLE 3:** Summary of confirmed, potential, and putative prebiotic exopolysaccharides (EPS) and oligosaccharides, detailing their sources, target microbes, enzymatic degradation mechanisms (CAZymes/PULs), and physiological effects in clinical and experimental models.

Evidence level^a^	EPS source (origin)	Target microbe (selectivity)	Enzymatic mechanism (CAZymes/PULs)	Experimental model	Physiological outcome	References
**Category A: Confirmed Prebiotics (Clinical evidence)**	Inulin (plant/fungal)	*Bifidobacterium* spp., *Faecalibacterium prausnitzii*	Inulinase (GH32); cross‐feeding on acetate/lactate	Human clinical studies	Bifidogenic effect; increased butyrate; improved bowel regularity and mineral absorption	S. Fan et al. (2024, 2023)
	Fructo‐oligosaccharides (FOS) (plant/fungal)	*Bifidobacterium* spp.	β‐Fructosidases (GH32)	Human clinical studies	Strong bifidogenic effect; increased SCFA production	S. Fan et al. (2024, 2023)
	Galacto‐oligosaccharides (GOS) (enzymatic)	*Bifidobacterium* spp., *Lactobacillus* spp.	β‐Galactosidases (GH2, GH42)	Human clinical studies (esp. infant)	Strong bifidogenic effect; pathogen exclusion; immune modulation	S. Fan et al. (2024, 2023)
	Resistant starch (RS2, RS3)	*Ruminococcus bromii* (keystone), *Eubacterium rectale*	Amylases (GH13); starch‐specific PULs	Human clinical studies	Increased butyrate production; improved insulin sensitivity; modulated satiety	Ze et al. (2013)
	Xylo‐oligosaccharides (XOS) (plant)	*Bifidobacterium* spp. (highly selective)	Xylanases (GH10, GH11)	Human clinical studies	Bifidogenic effect at low doses; increased SCFA	S. Fan et al. (2024, 2023)
	*Weissella confusa* VP30 (heteropolysaccharide)	Increased *Bacteroidetes* and *Prevotella*; reduced *Firmicutes*	Fermentation (CAZymes implied)	Human clinical study	Ameliorated functional constipation; increased defecation frequency/volume	Jin et al. (2023)
**Category B: Potential prebiotics (In vitro/in vivo models)**	Levan (*Paenibacillus*)	*Bacteroides thetaiotaomicron*	Endo‐levanase (GH32 family)	Mouse model	Increased butyrate; reduced inflammation	Rana and Upadhyay (2020)
	Kefiran (*Lactobacillus kefiranofaciens*)	*Bifidobacterium* spp., *Lactobacillus* spp.	β‐Galactosidase; extracellular hydrolases	In vitro fecal batch culture	Stimulation of bacterial growth; SCFA production	Lynch et al. (2018b)
	*Weissella cibaria* PFY06	Increased *Roseburia* and *Oscillibacter*	Fermentation (CAZymes implied)	High‐fat diet (HFD) murine model	Decreased weight gain; increased SCFAs, GLP‐1, and PPY	Xie et al. (2023)
	Kefir grains (heteropolysaccharide)	Significantly enhanced *Akkermansia* spp.	Fermentation (CAZymes implied)	Mouse model	Decreased weight gain; reduced plasma VLDL‐cholesterol	Lim et al. (2017)
	*Lactiplantibacillus plantarum* WLPL09 (EPS‐09)	Increased *Firmicutes*; decreased *Prevotella* and *Akkermansia*	Fermentation and host immunomodulation	Melanoma‐bearing murine model	Inhibited tumor growth (42.5%); enhanced host antitumor immunity	Q. Wang et al. (2024)
	*Lacticaseibacillus rhamnosus*	Restored gut microbiota diversity	Fermentation; host: TLR4/MAPKs activation	Immunosuppressed murine model	Restored immune indices; regulated cytokines; promoted SCFA	L. Chen et al. (2024)
	*Morchella esculenta* (MEP 2) (fungal)	Modulated gut microbiota composition	Fermentation and α‐amylase/α‐glucosidase inhibition	Type 2 diabetes (T2DM) murine model	Ameliorated liver inflammation; reduced HbA1c	H. Wu, Chen, et al. (2023)
	*Lactobacillus acidophilus* YL01	Modulated specific bacterial groups	Fermentation (CAZymes implied)	High‐fat diet (HFD) murine model	Ameliorated obesity and insulin resistance; increased acetate and butyrate	C. Zhao, Xie, et al. (2025)
	*Lactobacillus plantarum* KX041	Increased *Akkermansia* and *Lachnospiraceae*	Fermentation; host: inhibited TLR4/NF‐κB	High‐fat diet (HFD) murine model	Mitigated weight gain; reduced systemic inflammation	Yue et al. (2025)
	*Streptococcus salivarius* EPS	Modulated gut microbiota	Fermentation (CAZymes implied)	High‐fat diet (HFD) murine model	Prevented diet‐induced obesity; increased SCFA production	Shimizu et al. (2025)
	*Enterobacter cloacae* Z0206	Modulated hepatic enzymes (microbiota implied)	Fermentation; host: AMPK/Sirt1 activation	T2DM murine model	Hypoglycemic and hypolipidemic effects; improved insulin sensitivity	Huang et al. (2015)
	*Limosilactobacillus fermentum* Lf2	Modulated gut microbiota (e.g., *Peptococcaceae*)	Fermentation (CAZymes implied)	Murine (mouse) model	Reduced intestinal pro‐inflammatory cytokines; increased fecal SCFAs	Ale et al. (2025)
**Category C: Putative candidates (Techno‐functional only)**	*Leuconostoc citreum*‐BMS	None reported (techno‐functional)	N/A (emulsifier)	In vitro (emulsion study)	Stabilized oil‐in‐water emulsions at pH 3 and 7	Abid et al. (2021)
	*Neorhizobium urealyticum*	None reported (techno‐functional)	N/A (emulsifier)	In vitro (emulsion study)	High water/oil holding; pH‐stable emulsification	Roychowdhury et al. (2021)
	*Cryptococcus laurentii* 70766 (Fungal)	None reported (techno‐functional)	N/A (gelling agent)	In vitro (biomedical)	Formed cytocompatible hydrogels for tissue engineering	Hamidi et al. (2022)
	*Alteromonas infernus*	None reported (techno‐functional)	N/A (gelling agent)	In vitro (biomedical)	Formed microgels for controlled BMP‐2 drug delivery	Gélébart et al. (2022)
	*Enterococcus faecium* MC‐5	None reported (techno‐functional)	N/A (edible film component)	In vitro (food packaging)	Formed active antimicrobial film with bacteriocin	Tilwani et al. (2025)

Abbreviation: SCFA, short‐chain fatty acids.

^a^ Evidence Level refers to the categories defined in Box 2.

#### 3.2.1 The Microbial Enzymatic Arsenal: Carbohydrate‐Active Enzymes (CAZymes)

The human genome comprises a restricted set of about 17 CAZymes for the digestion of simple carbohydrates (Glowacki and Martens 2020). In contrast, the collective genome of the gut microbiota provides a substantial array of enzymes, allocating 1%–5% of its genes to carbohydrate metabolism, thereby greatly enhancing the digestive functions of the host. The breakdown of prebiotic fibers is mainly facilitated by four primary classes of microbial CAZymes. Glycoside hydrolases (GHs) represent the largest class of enzymes, comprising over 173 families, and play a vital role in the hydrolysis of glycosidic bonds between sugar monomers, exhibiting high substrate specificity (Glowacki and Martens 2020). Polysaccharide lyases (PLs) utilize a non‐hydrolytic elimination mechanism to cleave polysaccharides that contain uronic acids, such as pectin. Carbohydrate esterases (CEs) serve as critical enzymes that facilitate access to the primary polysaccharide structure by cleaving side‐chain modifications, including acetyl or feruloyl groups, thereby allowing other enzymes such as GHs and PLs to act effectively. Finally, non‐catalytic carbohydrate‐binding modules (CBMs) serve as essential accessory proteins that improve degradation efficiency by linking catalytic enzymes to their specific carbohydrate substrates (Glowacki and Martens 2020; Payling et al. 2020).

#### 3.2.2 Polysaccharide Utilization Loci (PULs): The Genetic Blueprint for Degradation

Glycan‐degrading bacteria, especially those in the Gram‐negative *Bacteroidetes* phylum, organize the genes for their enzymatic tools into physically clustered, co‐regulated cassettes referred to as PULs (S. Fan et al. 2023; Payling et al. 2020). The PULs exemplify an efficient evolutionary strategy, offering a comprehensive genetic framework for the targeting of a specific polysaccharide. The starch utilization system (Sus) in the gut symbiont *Bacteroides thetaiotaomicron* serves as the canonical model for PUL function. This system operates through a sequential process: Surface glycan‐binding proteins (SusD) first capture starch, which is subsequently cleaved into smaller oligosaccharides by an outer membrane‐anchored α‐amylase (SusG). These fragments are imported into the periplasmic space by a specialized TonB‐dependent transporter (SusC), with the gene physically linked to SusD, a defining characteristic of PULs (Figure 4B) (Glowacki and Martens 2020).

In the periplasm, a distinct group of GHs (SusA and SusB) facilitates the degradation to glucose monomers, regulated by a sensor‐regulator (SusR) that activates the transcription of the PUL solely in the presence of the target substrate. A single *Bacteroides* species can exhibit about 100 distinct PULs, providing significant metabolic flexibility. The “one PUL, one discrete structure” paradigm establishes a lock‐and‐key mechanism that underpins the molecular foundation of prebiotic specificity, permitting only those microbes possessing the appropriate PUL to degrade a particular EPS structure (S. Fan et al. 2023; Glowacki and Martens 2020; Payling et al. 2020).

#### 3.2.3 Diverse Degradation Strategies

The PUL system is characteristic of *Bacteroides*; however, other significant gut phyla have developed unique strategies. The *Bacteroides* PUL system, characterized by its complex mechanism of surface binding and periplasmic sequestration, exemplifies a fundamentally “selfish” strategy for nutrient acquisition (Glowacki and Martens 2020). By sequestering a polysaccharide and promptly importing the resultant breakdown products into its exclusive environment, the bacterium guarantees that it exclusively benefits from the complete energetic yield, thereby inhibiting competitors from accessing the valuable intermediates.

Conversely, numerous members of the *Firmicutes* phylum utilize more collaborative strategies. Certain organisms secrete their CAZymes into the extracellular environment, releasing simple sugars that serve as “public goods” accessible to the entire community. Certain specialist *Firmicutes*, such as *Ruminococcus* species, form extensive extracellular multienzyme complexes known as cellulosomes. These structures attach to the bacterial cell wall and localize a potent mixture of degradative enzymes onto insoluble fibers such as cellulose, representing an effective method for degrading resilient substrates. The distinction in molecular architecture—selfish sequestration compared to cooperative secretion—clarifies the various ecological niches inhabited by the predominant phyla in the gut (Ballan et al. 2020; Cantu‐Jungles and Hamaker 2020; Glowacki and Martens 2020; Illiano et al. 2020).

### 3.3 Step 3: Microbiota Modulation—The Ecological Dynamics of Fermentation

The introduction of a fermentable prebiotic into the gut ecosystem triggers a series of intricate ecological interactions, encompassing competition, cooperation, and extensive cross‐feeding networks that facilitate the distribution of metabolic energy within the microbial community. The initial degradation of a complex prebiotic is conducted by a specific group of primary degraders that possess the necessary PULs or secreted enzymes for the initial cleavage of the polymer (Glowacki and Martens 2020). In specific instances, a single keystone species is so effective in this role that its presence becomes essential for the community to obtain the fiber's nutrients. *Ruminococcus bromii* serves as a classic example of a keystone degrader of resistant starch (Ze et al. 2013). Its absence notably hinders the gut community's ability to ferment this substrate, illustrating the critical role specific microbes play in accessing nutrient sources for the entire ecosystem.

A fundamental ecological principle influencing prebiotic fermentation is metabolic cross‐feeding, also known as syntrophy, in which the byproducts produced by one microbial species act as essential nutrients for another. This metabolic transfer creates complex food webs that optimize energy extraction from the primary substrate and facilitate its efficient distribution within the community. The synthesis of butyrate, an essential SCFA for colonic health, exemplifies a critical microbial cross‐feeding cascade (Figure 4C). This process demonstrates that the initial breakdown of products from primary degraders are retained and subsequently utilized by other significant metabolic specialists within the ecosystem (Belenguer et al. 2006; Culp and Goodman 2023).

The synthesis of butyrate exemplifies microbial collaboration facilitated by an ecological division of labor. The process is initiated with primary fermenters, including *Bifidobacterium* species, which are stimulated by FOS and GOS (Belenguer et al. 2006). These fermenters effectively convert the prebiotics into lactate and acetate, although they generally do not produce butyrate. A specific group of specialized bacteria, mainly from the Firmicutes phylum, including notable species such as *Faecalibacterium prausnitzii*, *Eubacterium hallii*, and *Roseburia intestinalis*, engage in secondary fermentation (Louis and Flint 2017). These organisms, typically incapable of degrading the original complex prebiotic, are well‐adapted to utilize lactate and acetate produced by primary fermenters, converting them into butyrate. This specialization represents an effective evolutionary strategy, as it circumvents the metabolic costs associated with sustaining the genetic mechanisms for both polysaccharide degradation and highly efficient butyrate production within a single organism, thereby enhancing the resilience and metabolic capacity of the entire community (Belenguer et al. 2006; Culp and Goodman 2023; Louis and Flint 2017).

### 3.4 Step 4: Production of Bioactive Metabolites—The Language of Host‐Microbe Communication

The ultimate health benefits of proven prebiotic consumption are facilitated by bioactive microbial metabolites generated during fermentation, serving as the main mode of communication between the gut ecosystem and the host. SCFAs are the primary end products of anaerobic carbohydrate fermentation. The three predominant SCFAs—acetate (C2), propionate (C3), and butyrate (C4)—account for more than 95% of the total SCFA pool and are generally found in the colon in a molar ratio of about 3:1:1 (Procházková et al. 2023). The profile of SCFAs produced is contingent upon the substrate, resulting in a unique “metabolic signature” for each prebiotic that can be utilized for specific functional health outcomes.

The biosynthesis pathways for the primary SCFAs are distinct and executed by various members of the microbiota. Acetate, the predominant SCFA, is a widely produced fermentation product synthesized by various bacteria, such as *Bifidobacterium*, *Lactobacillus*, *Bacteroides*, and *Akkermansia* (Y. Fan and Pedersen 2021; Payling et al. 2020). This synthesis primarily occurs from pyruvate through the acetyl‐CoA pathway or via acetogens utilizing the Wood–Ljungdahl pathway. Propionate synthesis occurs through three primary pathways: The predominant succinate pathway utilized by *Bacteroidetes*, the acrylate pathway that converts lactate, and the propanediol pathway employed by species such as *Akkermansia muciniphila* for the fermentation of deoxy‐sugars. Butyrate production is primarily conducted by a specific group of anaerobic Firmicutes, including *F. prausnitzii* and *Roseburia* species. The synthesis initiates with the condensation of two acetyl‐CoA molecules, culminating in a crucial step involving the butyryl‐CoA:acetate CoA‐transferase pathway, which significantly utilizes acetate, thereby establishing a direct biochemical and stoichiometric foundation for the significance of microbial cross‐feeding in its production (Cantu‐Jungles and Hamaker 2020; Nogal et al. 2021; Payling et al. 2020; Procházková et al. 2023). Table 4 compares the biosynthesis, functions, and distinct physiological roles of the three primary SCFAs in the gut and systemic physiology.

**TABLE 4:** A comparative summary of the biosynthesis and diverse functions of the three primary short‐chain fatty acids (SCFAs), emphasizing their unique contributions to local gut health and systemic physiology (Bedu‐Ferrari et al. 2022; Louis and Flint 2017; Nogal et al. 2021; Payling et al. 2020).

SCFA	Key microbial producers	Key biosynthesis pathway	Primary local functions (gut)	Primary systemic functions (host)
Acetate (C2)	Broadly produced: *Bifidobacterium*, *Lactobacillus*, *Bacteroides*, *Prevotella*, etc.	From acetyl‐CoA; Wood–Ljungdahl pathway	Key cross‐feeding substrate for butyrate producers; systemic energy source	Substrate for peripheral lipogenesis and hepatic cholesterol synthesis
Propionate (C3)	Primarily *Bacteroidetes*; also, *Akkermansia*, *Roseburia*	Succinate pathway (dominant); acrylate pathway; propanodiol pathway	Minor energy source for colonocytes; pathogen inhibition via pH disruption	Substrate for hepatic gluconeogenesis; signals via GPR41/43 to stimulate satiety hormones (GLP‐1/PYY)
Butyrate (C4)	Specialist *Firmicutes*: *Faecalibacterium*, *Roseburia*, *Eubacterium*	Butyryl‐CoA:acetate CoA‐transferase	Primary energy source for colonocytes; enhances barrier integrity; potent HDAC inhibitor; anti‐inflammatory	Systemic anti‐inflammatory effects; key modulator of immune tolerance (T_reg_ induction); influences gut‐brain axis

Abbreviation: HDAC, histone deacetylase.

### 3.5 Step 5: Mediation of Host Health Benefits—The Systemic Impact of SCFAs

**FIGURE 5:**
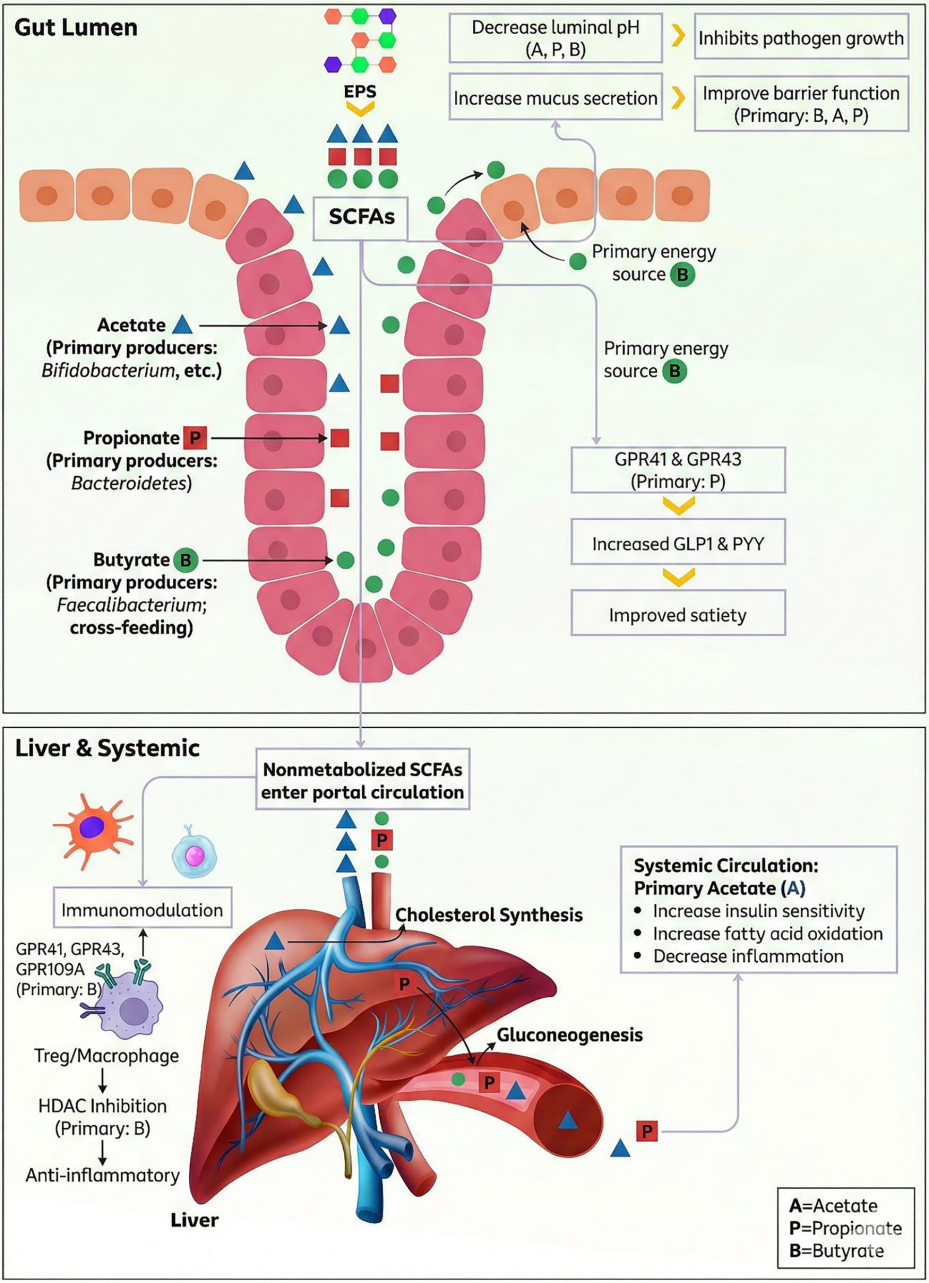
Local and systemic effects of SCFAs derived from EPS. Fermentation in the local environment produces acetate, propionate, and butyrate, which decrease luminal pH, enhance mucus secretion, and provide energy for colonocytes. These metabolites systematically regulate hepatic cholesterol and glucose metabolism while modulating immunity through HDAC inhibition. Propionate specifically activates GPR41/43, leading to the release of satiety hormones such as GLP‐1 and PYY, whereas acetate improves insulin sensitivity and promotes fatty acid oxidation. EPS, exopolysaccharide; SCFAs, short‐chain fatty acids. *Source*: Figure was based on Ross et al. (2024) and created in Canva.com with some icons from BioRender.com.

Upon production, SCFAs are immediately absorbed by the host and demonstrate a variety of significant biological effects via multiple mechanisms. They are not merely nutrients; rather, they represent a complex category of signaling molecules that function through a dual‐receptor and epigenetic mechanism, facilitating multilayered regulation of host physiology. Table 5 describes the main pathways by which SCFAs benefit gut health, whereas Figure 5 illustrates their effects on both gut and systemic health.

**TABLE 5:** The main pathway through which short‐chain fatty acids (SCFAs) exert their beneficial effects on gut health (Glowacki and Martens 2020; Nogal et al. 2021; Payling et al. 2020; Perler et al. 2025; Ross et al. 2024).

SCFA effect on gut health	Mechanism	Key SCFAs involved
Lowering intestinal pH and inhibiting pathogen growth	Direct release of H^+^ ions; consumption of luminal oxygen (creating anaerobic environment); direct antimicrobial effects; modulation of pathogen virulence factors via protein posttranslational modifications (e.g., acetylation and propionylation)	Acetate, propionate, butyrate
Providing energy to colonocytes	Butyrate is preferentially oxidized by colonocytes for ATP production via beta‐oxidation and Krebs cycle; supports colonocyte growth and metabolic activity; acetate and propionate contribute systemically	Butyrate (primary), acetate, propionate
Modulating immune responses	Activation of G‐protein coupled receptors (GPR41, GPR43, GPR109A) on immune and epithelial cells; inhibition of histone deacetylase (HDAC) leading to altered gene expression; reduction of pro‐inflammatory cytokines; promotion of anti‐inflammatory cytokines (e.g., IL‐10); Induction of T regulatory cells (T_regs_)	Acetate, propionate, butyrate (butyrate most potent HDAC inhibitor)
Improving gut barrier integrity	Enhancing tight junction assembly and function (via AMPK activation, GPCR signaling, upregulation of TJ proteins like claudin‐1, ZO‐1, occludin); promoting mucin production and secretion of antimicrobial peptides; stabilizing hypoxia‐inducible factor (HIF)	Butyrate (primary), acetate, propionate

Abbreviations: ATP, adenosine triphosphate; GPCR, G‐protein coupled receptors; TJ, tight junctions.

#### 3.5.1 Lowering Intestinal pH and Inhibiting Pathogen Growth

SCFAs promote a healthy gut environment through a multifaceted approach that extends beyond mere pH reduction. The acidic properties of SCFAs decrease the pH of the colonic lumen, establishing conditions that inhibit the proliferation of various pH‐sensitive pathogens, including *Salmonella* and *E. coli* (Nogal et al. 2021). The anti‐pathogen effect, however, constitutes an active process of environmental engineering. Butyrate metabolism in colonocytes enhances oxygen consumption, thereby contributing to the maintenance of a strictly anaerobic environment within the gut lumen. This process inhibits the proliferation of aerobic pathogens and promotes the growth of beneficial obligate anaerobes. Moreover, SCFAs can have direct antimicrobial effects. Propionate, for instance, can disrupt the intracellular pH homeostasis of *Salmonella typhimurium*, thereby inhibiting its proliferation. In contrast, butyrate can directly attenuate the virulence of enteric pathogens by downregulating the expression of key virulence genes, including those in *Salmonella* Pathogenicity Island 1 (SPI‐1), through protein posttranslational modifications (Nogal et al. 2021; Ross et al. 2024).

#### 3.5.2 Providing Energy to Colonocytes

Butyrate functions as the principal energy source for colonocytes, which are the epithelial cells that line the intestine. These cells primarily utilize butyrate through beta‐oxidation and the citric acid cycle to produce ATP, despite the presence of alternative substrates such as glucose (Perler et al. 2025; Ross et al. 2024). This metabolic support is essential for the health, proliferation, and function of the intestinal lining, significantly influencing mucus production and the maintenance of gut barrier integrity. Butyrate is primarily utilized locally, whereas acetate and propionate are absorbed into the bloodstream, playing a role in systemic energy metabolism within the liver and peripheral tissues (Ross et al. 2024).

#### 3.5.3 Modulating Immune Responses

SCFAs serve as essential mediators in communication between gut microbiota and the host's innate and adaptive immune systems, primarily via the activation of specific G‐protein coupled receptors (GPCRs) (Rekha et al. 2024). These receptors are present on the surface of diverse immune and epithelial cells. Their interaction with SCFAs initiates intracellular signaling pathways that typically enhance immune tolerance and reduce inflammation. The primary receptors in this pathway are GPR43 (FFAR2), which is preferentially activated by acetate and propionate; GPR41 (FFAR3), which primarily responds to propionate and butyrate; and GPR109A (HCA2), the specific receptor for butyrate. The specific affinities of these receptors for various SCFAs enable precise regulation of host immune responses according to the metabolic products of the gut microbiota (Ross et al. 2024).

SCFAs influence the immune system via epigenetic mechanisms, particularly by blocking histone deacetylase (HDAC) enzymes, in addition to receptor‐mediated communication (Simpson et al. 2023). Particularly, butyrate is a strong HDAC inhibitor that causes hyperacetylation of histones, changing chromatin structure and gene expression. Because it suppresses the production of pro‐inflammatory cytokines like TNF‐α and IL‐6 while concurrently promoting the transcription of anti‐inflammatory genes, including those for the cytokine IL‐10, this epigenetic regulation is an effective technique for managing inflammation. Additionally, by causing naïve T cells to differentiate into T regulatory cells (T_regs_), butyrate's capacity to increase histone H3 acetylation at the Foxp3 locus—the master transcriptional regulator of T_reg_ development—makes this mechanism essential for forming adaptive immunity (Illiano et al. 2020; Ross et al. 2024; Simpson et al. 2023).

#### 3.5.4 Improving Gut Barrier Integrity

SCFAs, particularly butyrate, play a vital role in preserving and improving the integrity of the gut barrier. Epithelial tight junctions (TJs) are promoted for their role in regulating paracellular permeability and inhibiting the translocation of harmful microorganisms and endotoxins, such as lipopolysaccharide (LPS), from the intestinal lumen into the bloodstream (Nogal et al. 2021; Ross et al. 2024). The mechanisms are multifaceted, involving the activation of AMP‐activated protein kinase (AMPK), which enhances the assembly of essential TJ proteins such as ZO‐1 and occludin, along with the positive regulation of TJ protein expression via GPCR signaling. In addition to their direct effects on TJs, SCFAs enhance barrier function by promoting the secretion of protective mucus and antimicrobial peptides (Simpson et al. 2023).

## 4 The EPS‐Microbiota‐Host Axis for Systemic Health

The elucidation of the systemic health benefits of microbial EPS necessitates a clear differentiation between “prebiotic” and “bioactive” mechanisms. A prebiotic effect, as defined by ISAPP, must be mediated by microbiota. Many EPS molecules demonstrate direct “bioactive” effects by interacting with host receptors or enzymes, which do not strictly conform to the prebiotic definition. EPS operates via a dual‐pathway framework: the direct “structural” pathway (bioactive) and the indirect “microbiota‐dependent” pathway (prebiotic). The primary prebiotic mechanism is defined by the EPS‐microbiota‐host axis, where indigestible EPS serves as a precision substrate for colonic fermentation. This process reshapes the gut ecology and generates key effector molecules—predominantly SCFAs—which act as ligands for GPCRs (GPR41/FFAR3 and GPR43/FFAR2) to initiate beneficial signaling cascades (Rekha et al. 2024). Concurrently, the unique physicochemical properties of EPS—charge density, Mw, and monosaccharide composition—allow for direct interactions with host enzymes, immune receptors, and pathogens (direct bioactivity). This section synthesizes these pathways to provide a conceptual analysis of how EPS orchestrates metabolic, immunological, and antimicrobial homeostasis.

### 4.1 Orchestration of Metabolic Homeostasis

Microbial EPS shows therapeutic potential for metabolic syndrome by concurrently addressing obesity, hyperglycemia, and dyslipidemia. Recent findings indicate that EPS functions as a multi‐target regulator of energy metabolism, integrating nutrient absorption, hormonal signaling, and hepatic lipid handling, rather than acting through isolated pathways.

The modulation of glucose and lipid metabolism initiates in the upper gastrointestinal tract through direct physicochemical interference. The inhibition of carbohydrate‐hydrolyzing enzymes, particularly α‐amylase and α‐glucosidase, represents a significant antidiabetic mechanism. Research on EPS derived from various LAB and *Enterococcus faecalis* indicates inhibition rates surpassing 90%, which effectively mitigates postprandial hyperglycemia by prolonging the degradation of complex carbohydrates (Ayyash, Abu‐Jdayil, Itsaranuwat, et al. 2020; Ayyash, Abu‐Jdayil, Olaimat, et al. 2020). The enzyme‐inhibitory activity is enhanced by the ability of EPS to sequester dietary cholesterol and bile acids in the intestinal lumen. EPS inhibits reabsorption and enhances fecal excretion, causing the liver to redirect endogenous cholesterol towards de novo bile acid synthesis, which results in reduced circulating LDL levels (Bhat and Bajaj 2018, 2019). The “sequestration effect” is structurally dependent, with binding affinity frequently correlated to the rheological properties and charge of the polymer.

**FIGURE 6:**
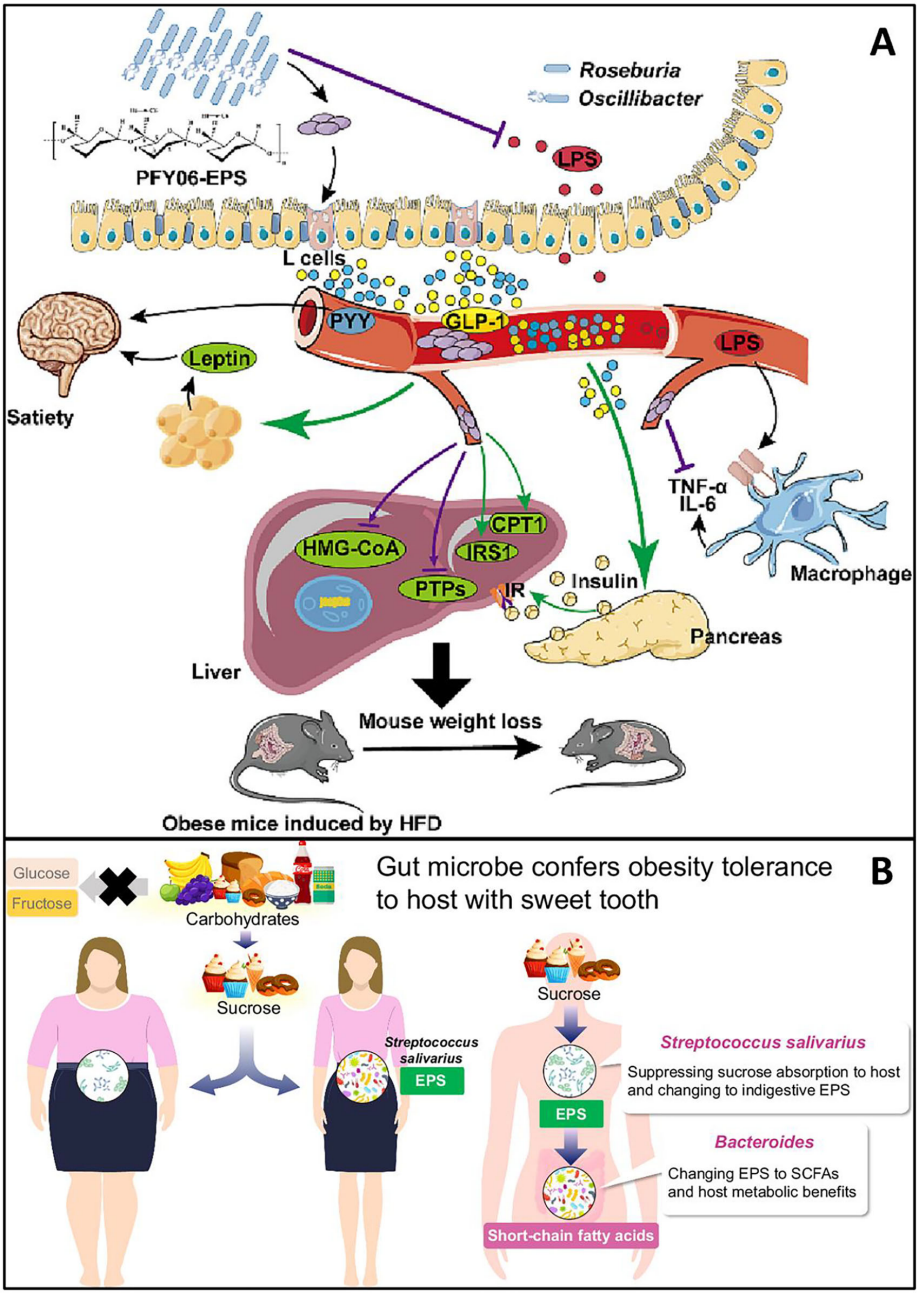
(A) Mechanistic diagram depicting the anti‐obesity effects of PFY06‐exopolysaccharide (EPS) derived from *Weissella cibaria* PFY06. This exopolysaccharide influences gut microbiota to increase short‐chain fatty acid (SCFA) production, thereby promoting the secretion of glucagon‐like peptide‐1 (GLP‐1) and peptide YY (PYY). PYY inhibits appetite through the central NPY2R pathway, whereas GLP‐1 and insulin modulate hepatic lipid metabolism. PFY06‐EPS concurrently inhibits the production of bacterial lipopolysaccharide (LPS), which reduces pro‐inflammatory cytokines (IL‐6, TNF‐α) and improves insulin resistance. (B) Proposed mechanism for the prevention of host obesity by sucrose‐preferring commensal microbes. This schematic demonstrates that excess dietary sucrose is fermented by bacteria, including *S. salivarius*, into EPS. These EPS subsequently regulate the EPS–SCFA–carbohydrate metabolism axis, thereby preventing diet‐induced obesity. EPS, exopolysaccharide. *Source*: (A) Reproduced from Xie et al. (2023) with permission from Elsevier Ltd. and (B) Figure was reprinted from Shimizu et al. (2025). Nature Communications. 2025. Licensed under CC BY 4.0.

In the gastrointestinal tract, the mechanism transitions from physical interaction to biological signaling through the gut–brain and gut–liver axes. For EPS variants that are fermentable, metabolism by specific colonic taxa, such as *Roseburia* and *Oscillibacter*, produces propionate and acetate, which function as significant signaling molecules. In the context of obesity, SCFAs activate GPR41 and GPR43 on enteroendocrine L‐cells, leading to the release of the anorexigenic hormones glucagon‐like peptide‐1 (GLP‐1) and peptide YY (PYY) (Figures 5 and 6A,B) (Shimizu et al. 2025; Xie et al. 2023). This hormonal cascade has a primary effect on the hypothalamus, inducing satiety, while also enhancing insulin sensitivity and promoting the browning of white adipose tissue through UCP1 upregulation.

Crucially, the regulation of diabetes and obesity by EPS is fundamentally connected to the reduction of systemic inflammation and oxidative stress. For example, EPS derived from *Lactobacillus plantarum* KX041 and *Enterobacter cloacae* Z0206 have demonstrated the ability to inhibit the TLR4/NF‐κB inflammatory pathway while simultaneously activating the AMPK/Sirt1 signaling axis (Huang et al. 2015; Yue et al. 2025). The activation of AMPK is crucial, as it inhibits hepatic gluconeogenesis by downregulating glucose‐6‐phosphatase and promotes glycolysis through the upregulation of hexokinase, thereby addressing the energy imbalance at the cellular level (Mao et al. 2024; C. Zhao, Xie, et al. 2025). The interaction between direct enzymatic inhibitions and indirect metabolic signaling underscores EPS as a significant modulator of the gut–metabolic interface (Table 6).

**TABLE 6:** Antidiabetic effects of microbial exopolysaccharides and proposed mechanisms.

Microbial source	EPS composition	Key antidiabetic effects	Proposed mechanisms	References
*Sorangium cellulosum* NUST06	Glucose and mannose	Reduced serum glucose in diabetic (35.9%–41.4%) and healthy (27.3%–30.1%) mice	Activation of insulin receptors, enhancing peripheral insulin sensitivity, promoting insulin secretion	Ding et al. (2004)
*Enterobacter cloacae* Z0206	l‐fucose, d‐glucose, d‐galactose, d‐glucuronic acid and pyruvic acid	Reduced fasting blood glucose, improved oral glucose tolerance, decreased serum insulin, reduced TG, TC, LDL‐c	Upregulated GK, HSL, ATGL, CPT1‐α, Glut2; downregulated G6P, FAS; through AMPK‐ and Sirt1‐mediated effects	Huang et al. (2015)
*Lactobacillus plantarum* C70	Arabinose, mannose, glucose, and galactose	Potent in vitro α‐amylase (91.0%) and α‐glucosidase (91.7%) inhibition	Postulated to block active sites of enzymes	Ayyash, Abu‐Jdayil, Itsaranuwat, et al. (2020)
*Pediococcus pentosaceus* M41	Glucose, mannose, galactose, and arabinose	Pronounced in vitro α‐amylase (86.8%) and α‐glucosidase (90.8%) inhibition	Postulated to block active sites of enzymes; glycosidic linkages may play a role	Ayyash, Abu‐Jdayil, Olaimat, et al. (2020)
*Lactiplantibacillus plantarum* LS5 and LU5	Arabinose, mannose, glucose, and galactose	Concentration‐dependent α‐amylase inhibition (e.g., 34.43% for LS5‐EPS and 35.13% for LU5‐EPS)	Depends on origin, sugar composition, Mw, and concentration	Bagher Hashemi et al. (2022)
*Enterococcus faecalis* 84B	Arabinose and glucose	High in vitro α‐amylase (89.6%) and α‐glucosidase (90.0%) inhibition	Postulated to block active sites, alter allosteric sites, bind to substrates; involves glycosidic bonds, possibly a pseudo‐sugar ring	A. H. Ali et al. (2023)
*Lactiplantibacillus plantarum* MY04	Mannose	Enhanced insulin sensitivity in HepG2 cells; increased glucose consumption, glycogen synthesis; upregulated HKase, PKase activities; mitigated oxidative stress (increased SOD, CAT)	Upregulation of glycolytic pathway enzymes; mitigation of oxidative stress	Mao et al. (2024)
*Lacticaseibacillus rhamnosus* ACS5	Mannose, galactose, and *N*‐acetyl mannosamine	High in vitro α‐amylase inhibition (60%)	Inhibition of α‐amylase by binding to active site; reduced binding at higher concentrations due to steric hindrance or glycosidic bond hydrolysis	İnanan et al. (2024)
*Penicillium janthinellum* N29	α‐d‐Manopyranose and β‐d‐galactofuranose	Strong in vitro α‐glucosidase inhibition (70.52%); reduced blood glucose, improved glucose tolerance, increased insulin sensitivity, ameliorated dyslipidemia in vivo	α‐Glucosidase inhibition; associated with structural characteristics like α‐(1 → 4) glycosidic linkages and β‐d‐(1 → 6)‐glycosidic bonds	Shao et al. (2023)
*Morchella esculenta*	α‐d‐Glucopyranose, β‐d‐glucopyranose, and α‐d‐mannopyranose	Strong in vitro α‐amylase, moderate α‐glucosidase inhibition; ameliorated liver inflammatory infiltration, increased pancreas islet size, reduced serum HbA1c in vivo	α‐Amylase inhibition; gut microbiome modulation	H. Wu, Chen, et al. (2023)

Abbreviation: EPS, exopolysaccharides.

### 4.2 Cellular Defense and Immunological Surveillance

The biological function of bioactive EPS encompasses not only metabolic regulation but also the maintenance of cellular integrity and immune surveillance. A critical analysis of the literature indicates a functional convergence among antioxidant defense, immunomodulation (Table 7), and antitumorigenicity. Oxidative stress is a recognized precursor to inflammation and carcinogenesis; thus, the antioxidant capacity of EPS serves as a fundamental preventive mechanism.

**TABLE 7:** Immunomodulating activities of microbial exopolysaccharides.

Microbial source	EPS composition	Experimental models	Immunomodulatory effect and its mechanism	References
*Lactobacillus reuteri* Mh‐001	Mannose, glucosamine, rhamnose, glucose, and galactose. High in galactose (39%) and rhamnose (15%)	RAW 264.7 murine macrophage cell line	Showed the highest anti‐inflammatory effect among the unpurified EPS tested by significantly reducing TNF‐α secretion in LPS‐stimulated macrophages. The mechanism is linked to its high galactose and rhamnose content, which enhances its anti‐inflammatory properties	Y. C. Chen et al. (2019)
*Lactobacillus fermentum* Mh‐002	Mannose, glucosamine, rhamnose, glucose, galactose, and xylose	RAW 264.7 murine macrophage cell line	Demonstrated an anti‐inflammatory effect by reducing TNF‐α secretion in LPS‐stimulated macrophages, though it was less potent than the EPS from *L. reuteri* Mh‐001	
*Enterococcus faecalis* Mh‐003	Mannose, glucosamine, rhamnose, glucose, galactose, and xylose	RAW 264.7 murine macrophage cell line	Exhibited the least potent anti‐inflammatory effect of the three strains tested, but still reduced TNF‐α secretion compared to the control	
*Lactiplantibacillus plantarum* MM89	Heteropolysaccharide composed of glucose and mannose with an average molecular weight (Mw) of 138 kDa	In vitro: RAW 264.7 cells In vivo: Cyclophosphamide‐induced immunosuppressed mouse model	Possesses strong immunostimulatory activity In vitro, it increased phagocytosis, acid phosphatase activity, and cytokine production (IL‐10, IL‐6, IL‐1β, and TNF‐α) In vivo, it increased splenic indices, intestinal IgA levels, serum cytokines, and lymphocyte proliferation	Rajoka et al. (2022)
*Bifidobacterium longum* subsp. *longum* 35624	Negatively charged exopolysaccharide (EPS624) with a Mw of 639.9 kg/mol	Human peripheral blood mononuclear cells (hPBMCs) and human foreskin fibroblasts	Showed immunoregulatory properties by inducing a dose‐dependent IL‐10 secretion from hPBMCs, indicating an anti‐inflammatory response. It also induced a pro‐inflammatory TNF‐α response. The IL‐10 induction mechanism is suggested to be TLR‐2 dependent	Bagnol et al. (2023)
*Bacillus megaterium* DSKPDF CMST3	Composed of glucose, galactose, and rhamnose	Rohu (*Labeo rohita*) fingerlings	Acted as an immunostimulant in fish by enhancing immunological and hematological parameters, upregulating immune genes (IL‐10, IL‐1β, and TGFβ), and increasing survival against *Aeromonas hydrophila* infection	Sathishkumar et al. (2024)
*Lacticaseibacillus rhamnosus* B6	Heteropolysaccharide (F1) composed of rhamnose, glucose, and galactose (molar ratio 3.7:1.5:1) with a Mw of 1.577 × 10^6^ Da	RAW 264.7 murine macrophage cell line	Stimulated phagocytic activity, TNF‐α expression, and nitric oxide (NO) release. It skewed macrophage polarization towards the M1 phenotype through the activation of the NF‐κB signaling pathway	Han et al. (2024)
*Lacticaseibacillus rhamnosus* ZFM216	Composed of 10 different monosaccharides, including uronic acids, with a Mw of 19.9 kDa	In vitro: RAW 264.7 cells In vivo: cyclophosphamide‐induced immunosuppressed mouse model	In vitro, it promoted the release of NO, TNF‐α, IL‐1β, and IL‐6. The mechanism involves the activation of TLR4 and MAPKs signaling pathways In vivo, it restored body weight, immune organ indices, and regulated cytokines (TNF‐α, IFN‐γ, IL‐10) by modulating gut microbiota and promoting SCFA production	L. Chen et al. (2024)
*Chaetomium globosum* CGMCC 6882	Heteropolysaccharide (CGP‐TS) composed of rhamnose, arabinose, galactose, glucose, and xylose with a Mw of 613.235 kDa	RAW 264.7 murine macrophage cell line	Exhibited immunomodulatory activity by enhancing phagocytic activity and promoting the release of cytokines (TNF‐α, IL‐1β, IL‐6, and NO)	S. Wang et al. (2025)
*Limosilactobacillus fermentum* Lf2	Composed of a high‐molecular‐mass β‐glucan (1.23 × 10^6^ g/mol) and two medium‐molecular‐mass polysaccharides (8.8 × 10^4^ g/mol)	C57BL/6 mice	Reduced pro‐inflammatory cytokines (TNF‐α, IFN‐γ, IL‐12, and IL‐6) in the intestines. The health‐promoting properties of the bacterium are suggested to be linked to its EPS production, which also increased fecal SCFAs and modulated gut microbiota	Ale et al. (2025)
*Bifidobacterium longum* subsp. *longum* XZ01	Neutral heteropolysaccharide (BLEPS‐1) composed of mannose, xylose, glucose, and galactose	In vitro: RAW 264.7 cells In vivo: DSS‐induced ulcerative colitis mouse model	A synbiotic of BLEPS‐1 and *Lactobacillus acidophilus* alleviated intestinal inflammation by reducing pro‐inflammatory cytokines and promoting M2 macrophage polarization. The effect is mediated by modulating gut microbiota and increasing SCFAs and aromatic amino acid metabolites, which, in turn, modulate colonic Tlr4/Ahr signaling pathways	Ma et al. (2025)
*Acetilactobacillus jinshanensis* BJ01	Mannose, xylose, and glucose with a MW of 156.58 kDa	In vitro experiments on the RAW 264.7 murine macrophage cell line	EPS‐1 demonstrated immunomodulatory activity by significantly activating RAW 264.7 macrophages, which enhanced the production of NO and pro‐inflammatory cytokines like IL‐6 and TNF‐α. This effect is mediated through the activation of the NF‐κB and MAPK signaling pathways	Tian et al. (2025)

Abbreviations: EPS, exopolysaccharides; SCFA, short‐chain fatty acids.

Microbial EPS reduces oxidative stress via a structure‐dependent mechanism involving scavenging and chelating processes. The presence of specific functional groups, including hydroxyl, carboxyl, and sulfate, facilitates the direct neutralization of reactive oxygen species (ROS) and the chelation of prooxidant transition metals such as Fe^2+^ (Ayyash, Abu‐Jdayil, Itsaranuwat, et al. 2020; M. Kumari et al. 2023). This activity inhibits oxidative damage to DNA and cellular lipids, thereby effectively interrupting the initial mutagenesis steps typically necessary for tumorigenesis. For example, EPS derived from *L. rhamnosus* ACS5 has demonstrated substantial protection against DNA damage and complete ABTS scavenging (İnanan et al. 2024).

When prevention is ineffective and tumorigenesis develops, EPS demonstrates antitumor effects via distinct direct and indirect pathways. Direct mechanisms encompass the induction of apoptosis and the arrest of the cell cycle. Highly sulfated or specifically branched EPS molecules can induce mitochondrial dysfunction or activate the caspase cascade (caspase‐3, ‐8, ‐9) in cancer cells, resulting in G0/G1 phase arrest (Chakraborty et al. 2019; J. Wu et al. 2021). Experimental models utilizing EPS from *P. aeruginosa* and *Weissella cibaria* have demonstrated dose‐dependent cytotoxicity and necrosis in colorectal and cervical cancer cell lines (Du et al. 2023; Tahmourespour et al. 2020).

**FIGURE 7:**
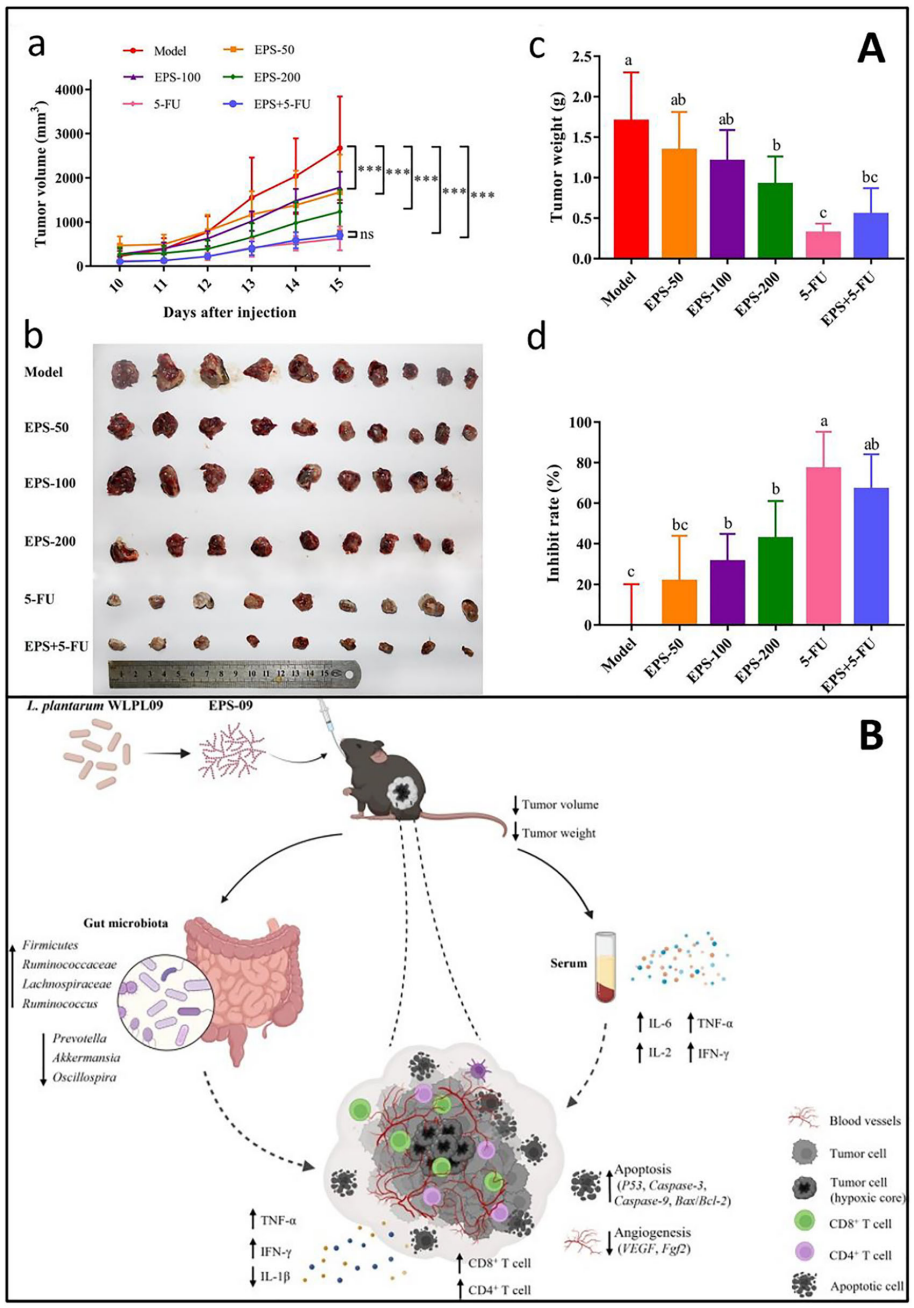
(A) The inhibitory effect of EPS‐09 from *Lactobacillus plantarum* WLPL09 on B16F10 melanoma tumor growth in mice: (a) tumor growth curves, (b) representative photographs of excised tumors on Day 15, (c) final tumor weight on Day 15, and (d) tumor growth inhibition rate. Data are presented as mean ± SD. ***p < 0.001 compared to the Model group. Groups labeled with different letters are significantly different from one another (*p* < 0.05). (B) Schematic overview of the antitumor mechanisms of EPS‐09 in melanoma‐bearing mice. EPS‐09 administration inhibits tumor growth by (I) enhancing host antitumor immunity via promotion of lymphocyte proliferation, increased tumor infiltration of CD4^+^ and CD8^+^ T cells, and elevated levels of key cytokines (e.g., TNF‐α, IFN‐γ, and IL‐2); (II) inducing tumor cell apoptosis through the upregulation of p53, caspase‐3, and caspase‐9, and an increased Bax/Bcl‐2 ratio; (III) suppressing angiogenesis by downregulating VEGF and FGF2 expression; and (IV) modulating the gut microbiota, marked by an increase in Firmicutes and a decrease in *Prevotella* and *Akkermansia*. This specific microbial profile shift (increase in Firmicutes; decrease in *Akkermansia*/*Prevotella*) is a context‐dependent finding associated with the observed antitumoral efficacy in the B16F10 melanoma model. This finding differs from general metabolic health contexts where *Akkermansia muciniphila* is often considered beneficial for SCFA (specifically acetate) production and mucosal integrity. However, in this *cancer* model, the therapeutic effect is strongly correlated with a shift towards butyrate‐producing Firmicutes (e.g., *Ruminococcaceae* and *Lachnospiraceae*), which are directly linked to enhanced antitumor immunity and T‐cell infiltration, as detailed in the text (Section 4.2). EPS, exopolysaccharide; SCFAs, short‐chain fatty acids. *Source*: Figure is reprinted from Q. Wang et al. (2024) with permission from Elsevier Ltd.

The primary antitumor mechanism of physiological importance is likely indirect, facilitated by the modulation of the host immune system. In this context, EPS functions primarily as a signaling molecule rather than a prebiotic substrate, commonly termed a “bioactive biopolymer” or “biological response modifier.” Specific EPS variants can modulate inflammation through interactions with pattern recognition receptors (PRRs), including toll‐like receptors (TLRs), by reducing TNF‐α levels or acting as immunostimulants to strengthen host defense mechanisms (Y. C. Chen et al. 2019; Rajoka et al. 2022). EPS acts as a biological response modifier and is often referred to as a pathogen‐associated molecular pattern (PAMP). This recognition activates the immune system, improving macrophage phagocytosis, promoting splenic lymphocyte proliferation, and altering the cytokine environment. This modulation exhibits a dichotomous and context‐dependent nature: Certain EPS variants, including those from *Lactobacillus reuteri*, suppress inflammation by lowering TNF‐α, whereas others, such as those from *Lactiplantibacillus plantarum*, function as immunostimulants to enhance defense against immunosuppression (Y. C. Chen et al. 2019; Rajoka et al. 2022). In cancer, this act leads to the infiltration of CD4^+^ and CD8^+^ T cells into the tumor microenvironment and the upregulation of pro‐inflammatory cytokines (TNF‐α and IFN‐γ) essential for tumor rejection (Figure 7A,B) (Q. Wang et al. 2024).

Furthermore, fermentable EPS variants function as prebiotics, influencing gut microbiota to promote beneficial bacteria such as *Firmicutes*, *Ruminococcaceae*, *Lachnospiraceae*, and *Ruminococcus*, while diminishing pro‐inflammatory or pro‐carcinogenic microbes including *Prevotella*, *Akkermansia*, and *Oscillospira*. (It is necessary to note that *Akkermansia* is typically linked to favorable metabolic results; however, its reduction in this cancer model correlates positively with an increase in butyrate‐producing *Firmicutes*, which play a significant role in enhancing antitumor immunity). This modulation can result in the production of SCFAs, such as butyrate, which exhibit anticancer properties. Certain EPS may also indirectly inhibit tumor growth by suppressing angiogenesis via the downregulation of associated genes (e.g., VEGF and FGF2) (Chakraborty et al. 2019; J. Wu et al. 2021).

### 4.3 Antimicrobial/Antiviral Defense

The ultimate level of EPS functionality involves the direct inhibition of pathogenic invasion. The antimicrobial properties of EPS primarily involve direct molecular interference and competitive exclusion, in contrast to the metabolic and immunomodulatory effects that depend significantly on host signaling. This technique presents a crucial alternative approach in the context of rising antimicrobial resistance.

The antibacterial efficacy of EPS is characterized by its capacity to undermine bacterial structural integrity and interfere with communal behaviors. EPS from LAB and marine sources can disrupt peptidoglycan synthesis and cell division, resulting in lysis of both Gram‐positive and Gram‐negative pathogens (A. H. Ali et al. 2023; Raposo et al. 2014). The “antibiofilm” activity is more complex than basic bacteriostasis, as it involves the inhibition of initial pathogen adhesion or the disruption of quorum sensing signals necessary for virulence (Ghanaim et al. 2025). For example, EPS derived from *P. aeruginosa* AG01 has been demonstrated to inhibit biofilm formation in *Staphylococcus aureus* by as much as 98%, effectively removing the pathogen's primary defense mechanism (Ghanaim et al. 2025).

In the context of viral infections, EPS serves as a universal entry inhibitor. The structural characteristics of EPS, specifically sulfation and negative charge density, enable these polymers to imitate host cell surface receptors or directly interact with viral glycoproteins. Steric shielding inhibits the adsorption and entry of enveloped viruses, including HSV‐1/2, VSV, and adenovirus (Saad et al. 2024; Sánchez‐León et al. 2020). Recent studies on cyanobacterial sulfated HePSs have revealed a “triple‐threat” mechanism, which includes direct virucidal effects, inhibition of viral entry, and disruption of intracellular replication (Saad et al. 2024). EPS, in conjunction with its ability to enhance host antiviral immunity through the gut‐microbiota axis, establishes a robust barrier against microbial pathogenesis.

## 5 Techno‐Functional Properties of Microbial EPS

The industrial applicability of microbial EPS extends beyond their biological activities, grounded in their remarkable techno‐functional versatility. EPS, as natural and biodegradable macromolecules, are gaining recognition as sustainable alternatives to synthetic polymers in the food, pharmaceutical, and biomedical industries. Their utility is determined by their ability to modulate the rheological and structural properties of complex systems, including the stabilization of multiphase emulsions and the engineering of robust hydrogel networks and bioactive delivery vehicles. This section synthesizes the physicochemical mechanisms underlying these functionalities, categorizing them into two main domains: the structural engineering of multiphase systems and the development of advanced barrier and delivery architectures.

### 5.1 Structural Engineering of Multiphase Systems

The development of stable colloidal systems, including emulsions and hydrogels, poses a considerable challenge in material science, especially given the increasing need to substitute synthetic surfactants (e.g., Tweens and Spans) with biocompatible alternatives. Microbial EPS fulfills this requirement by acting as “structural engineers” at the interface and within the bulk phase of aqueous systems. Their efficacy in these functions is contingent upon specific molecular attributes, including Mw, branching configurations, and charge density, which affect their interactions with solvents, oils, and other biopolymers (McClements et al. 2017; Nejatian and Abbasi 2022).

#### 5.1.1 Interfacial Stabilization and Emulsification

Polysaccharides are generally hydrophilic; however, microbial EPS display a distinctive amphiphilic nature due to protein moieties or particular hydrophobic functional groups, enabling their adsorption at oil–water interfaces (Pourjafar et al. 2023). In contrast to small‐molecule surfactants that quickly reduce surface tension, EPS predominantly stabilize emulsions via steric hindrance and electrostatic repulsion, creating a dense, hydrated interfacial layer that inhibits droplet coalescence (Table 8).

**TABLE 8:** Emulsifying activities of microbial exopolysaccharides.

Microbial strain	Composition and MW	Key findings	References
*Bacillus mojavensis* (Bacteria)	Levan, MW: 2.3 MDa	*B. mojavensis* produced levan efficiently, achieving a yield of 22 g/L. The structure is made up of a main backbone of (β2 → 6)‐linked fructose, with noticeable branching (9%) at the (β2 → 1) positions. The emulsifying capacity of levan, at a 3% concentration, yielded a stable emulsion with a uniform distribution of finely dispersed droplets. Notably, levan outperformed xanthan gum in terms of droplet size reduction at the same concentration, indicating a high potential for efficient emulsification in food formulations	Haddar et al. (2021)
*Leuconostoc citreum‐*BMS (Bacteria)	A combination of α‐(1,6)‐dextran branched at the third position and β‐(2,6)‐levan. MW: 1.8 MDa	*L. citreum* produces an anionic EPS, a mixture of dextran and levan with phosphate groups, which effectively stabilizes emulsions. Its effectiveness is demonstrated by its high emulsifying activity, generation of small droplet sizes, and significant reduction of interfacial tension, all maintained across acidic (pH 3) and neutral (pH 7) pH conditions	Abid et al. (2021)
*Neorhizobium urealyticum* sp. (Marine bacteria)	A heteropolysaccharide composed of glucose and galacturonic acid. MW: 0.207 MDa	An amphiphilic EPS from *N. urealyticum*, composed of galacturonic acid and glucose, offers significant water‐holding, oil‐holding, and swelling capabilities. Its pH‐stable emulsifying properties and effective astaxanthin nanoencapsulation, with controlled release, suggest promising applications in food industries requiring enhanced stability and delivery	Roychowdhury et al. (2021)
*Lactobacillus plantarum* ATCC 8014 (Bacteria)	A mixture of mannose, galactose, fructose, and glucose	EPS from *L. plantarum* effectively emulsified eugenols, yielding stable nanoscale emulsions (192 ± 1.89 nm) with high zeta‐potential (−32 ± 1.90 mV). This EPS‐stabilized emulsion demonstrated prolonged stability (90 days) and significantly enhanced antimicrobial activity against foodborne pathogens, reducing microbial load on lettuce	Balyan et al. (2024)
*Lactiplantibacillus plantarum* PRK7 and *L. plantarum* PRK11	A mixture of glucose, galactose, xylose, and mannose	Both EPS‐7 and EPS‐11 showed emulsification activity with edible oils like coconut, sesame, almond, castor, and neem oil. EPS‐11 demonstrated higher emulsification activity than EPS‐7, particularly with coconut and castor oil. The results suggest that these microbial EPS could potentially replace synthetic polymers as emulsifiers in the food and cosmetic industries	Kowsalya et al. (2023)
*Tetragenococcus halophilus* SNTH‐8	Composed of arabinose, xylose, fucose, galactose, glucose, and glucuronic acid	Two EPS, THPS‐1 and THPS‐2, were isolated from *T. halophilus* SNTH‐8 revealing moderate emulsifying capacities. THPS‐2 demonstrated enhanced emulsification, attributed to its higher uronic acid content, rougher surface, and a highly branched, porous structure. Both EPSs demonstrated concentration‐dependent emulsification, maintaining reasonable stability even at higher concentrations. These findings suggest that THPS‐1 and THPS‐2 are promising natural emulsifiers for industrial applications	Yang et al. (2022)

Recent mechanistic studies emphasize the role of specific structural features in augmenting this stabilizing effect. For instance, the high‐Mw EPS (EPS‐BMS) derived from *Leuconostoc citreum*‐BMS, featuring an α‐(1,6)‐dextran backbone with branching at C3, illustrates that increased branching can improve steric coverage at the interface. This polymer demonstrated effective inhibition of droplet aggregation for more than 15 days across a wide pH range (3–7), indicating that its spatial configuration offers a strong physical barrier against environmental stress (Abid et al. 2021). Similarly, the Mw of the polymer is crucial in determining droplet size. A levan‐type EPS derived from *Bacillus mojavensis* (2.3 MDa) demonstrated the capability to produce notably smaller emulsion droplets compared to the commercial standard xanthan gum at equivalent concentrations (Haddar et al. 2021). The differing emulsion index highlights a structure–function relationship in which the specific linkage type—(β2 → 6)‐fructofuranosyl residues—promotes tighter interfacial packing.

In addition to providing physical stabilization, EPS can enhance the multifunctionality of emulsions. The development of eugenol oil‐in‐water nanoemulsions demonstrated that EPS from *L. plantarum* achieved kinetic stability for over 90 days and synergistically enhanced antimicrobial activity against pathogens such as *Listeria monocytogenes* (Balyan et al. 2024). This indicates that EPS‐stabilized emulsions function as active entities rather than passive delivery systems, demonstrating the ability to endure challenging processing conditions, such as high salinity and thermal fluctuations, while enhancing food safety.

#### 5.1.2 Hydrogel Network Formation

As functionality transitions from the interface to the continuous phase, EPS function as adaptable components for hydrogels—three‐dimensional networks that can retain significant amounts of water. The transition from a viscous solution to a gel state occurs through physical cross‐linking, such as hydrogen bonding and electrostatic interactions, or through chemical cross‐linking via covalent bonding. The selected mechanism determines the mechanical strength, biodegradability, and stimulus responsiveness of the gel, which is essential for applications in tissue engineering and food texture design (McClements 2024; Yin et al. 2023).

Chemical cross‐linking strategies have been utilized to develop durable biomedical scaffolds. For example, a tyramine‐conjugated EPS derived from *Cryptococcus laurentii* 70766 creates an enzymatically cross‐linked hydrogel with adjustable swelling ratios (Hamidi et al. 2022). The observed negative swelling ratio in these gels suggests a high cross‐linking density, crucial for preserving structural integrity in physiological environments. The properties of fungal EPS, along with its inherent cytocompatibility, establish it as a competitive alternative to alginate‐based systems in the field of regenerative medicine. In contrast, physical cross‐linking facilitates the formation of dynamic, stimuli‐responsive networks. The bacterial EPS “Infernan” (*Alteromonas infernus*) has been incorporated into calcium‐cross‐linked microgels for the purpose of bone regeneration (Gélébart et al. 2022). Through the modulation of network density via ionic interactions, these microgels can regulate the release kinetics of growth factors such as bone morphogenetic protein‐2 (BMP‐2), illustrating the potential of the polymer's anionic characteristics for targeted drug delivery.

In food systems, EPS typically operate within composite networks, engaging with proteins to alter texture. The incorporation of *Lactobacillus casei* EPS into acid‐induced soybean protein isolate (SPI) gels demonstrates the efficacy of electrostatic complexation (Ren et al. 2025). Under acidic conditions, negatively charged EPS interacts with positively charged protein domains, thereby reinforcing the matrix and diminishing water channels. This leads to a denser and more uniform microstructure, demonstrating the ability of EPS to function as textural modulators that address the functional limitations of plant‐based proteins.

### 5.2 Advanced Barrier and Delivery Systems

The physicochemical robustness of microbial EPS enables their use as barrier materials at various length scales, from macroscopic edible films that protect food surfaces to nanoscopic capsules that safeguard sensitive bioactives. The fundamental mechanism in both contexts is based on the polymer's capacity for self‐assembly or cross‐linking into cohesive matrices that control mass transfer (e.g., gases and moisture) and provide resistance to environmental degradation.

#### 5.2.1 Edible Films and Active Packaging

Edible films constitute a sustainable advancement in food preservation, utilizing EPS matrices as selective barriers to oxygen and moisture. The mechanical performance of these films is significantly influenced by formulation engineering, especially the interaction between plasticizers and polymers. Studies on kefiran films demonstrate that glycerol increases solubility, whereas polyols such as d‐glucitol are necessary to decrease microhardness and enhance flexibility (Montoille et al. 2021). The ability to adjust this property is essential for commercial implementation, enabling films to be tailored to meet specific packaging requirements.

Recent advancements have progressed from passive barriers to “active packaging” systems that utilize EPS matrices to incorporate antimicrobial agents. The incorporation of EPS from *Enterococcus faecium* MC‐5 into chitosan‐based composite films illustrates this synergy (Tilwani et al. 2025). The EPS enhances the structural density of the film, improving UV and water barriers, whereas concurrently delivering bacteriocins to inhibit pathogens such as *Listeria*. Similarly, ternary blends that include bacterial levan, pullulan, and chitosan leverage the unique properties of each polymer: Pullulan provides oxygen barrier capabilities, chitosan offers antimicrobial effects, and levan contributes to mechanical strength (Gan et al. 2022). These composites illustrate that EPS are most effective when integrated into synergistic polymer blends, resulting in multifunctional coatings that actively prolong shelf life.

#### 5.2.2 Encapsulation and Bioactive Protection

At the nanoscale, the self‐assembly and conjugation properties of EPS are utilized to encapsulate unstable bioactives, safeguarding them from gastric degradation and facilitating targeted release. This application is significantly dependent on the responsive characteristics of EPS, especially their sensitivity to pH levels.

**FIGURE 8:**
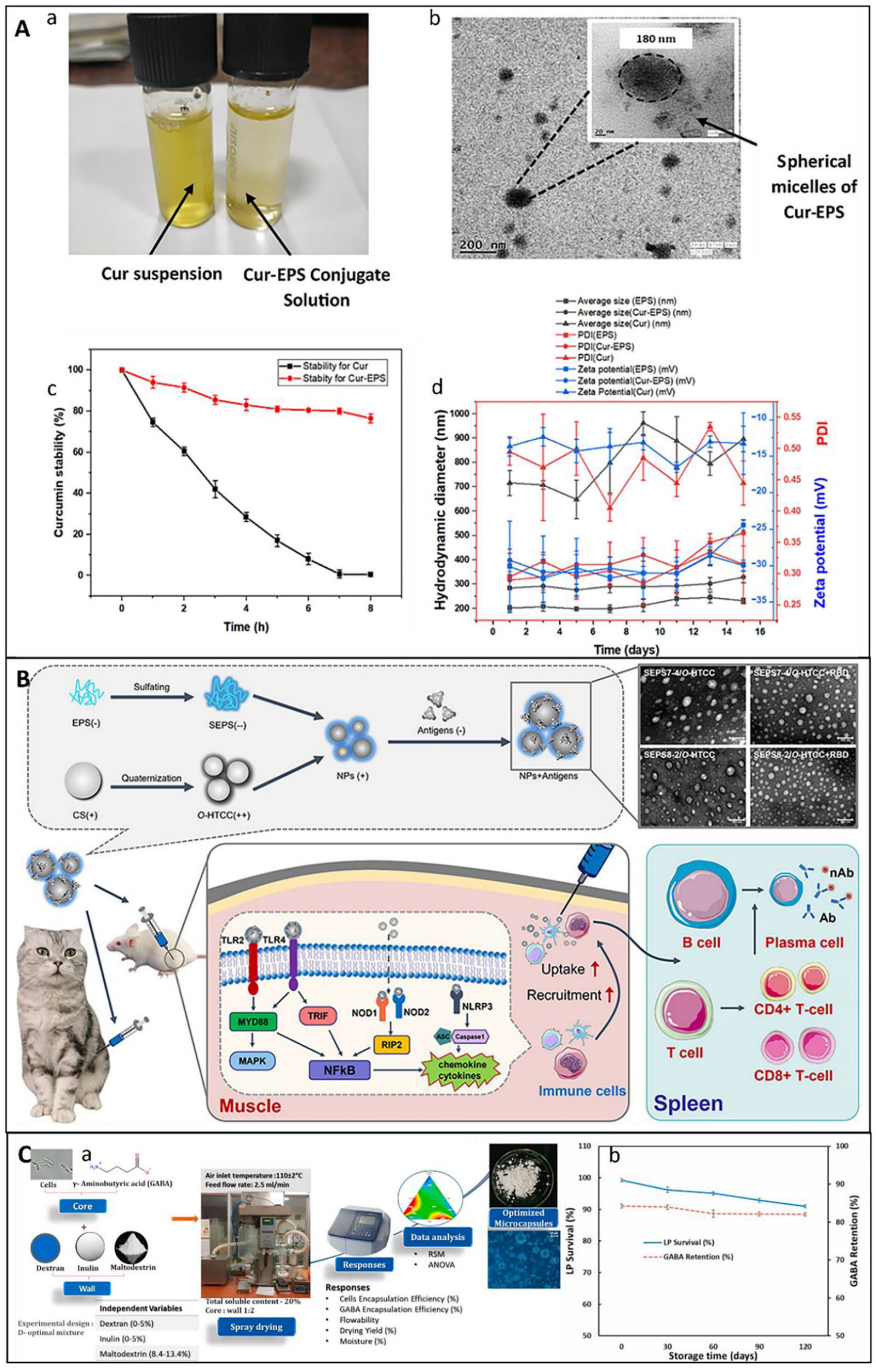
**(A**) Physicochemical characterization and stability of curcumin‐exopolysaccharide (Cur‐EPS) conjugates. (a) Comparison of a curcumin (Cur) suspension and an aqueous solution of Cur‐EPS conjugates derived from *Bacillus megaterium* RB‐05. (b) Transmission electron microscopy (TEM) image of Cur‐EPS micellar structures (inset scale bar = 20 nm). (c) Comparative stability of free Cur and Cur‐EPS micelles. (d) Storage stability of exopolysaccharide (EPS), Cur, and the Cur‐EPS conjugate evaluated by changes in zeta potential, average diameter, and polydispersity index (PDI). Data are expressed as mean ± standard error (*n* = 3). **(B**) Schematic of the mechanism for EPS‐based nanoparticle adjuvants. The positively charged nanoparticles, fabricated from modified *Lactobacillus* EPS and chitosan, effectively adsorb a SARS‐CoV‐2 antigen for sustained release and enhanced uptake by macrophages. The adjuvant stimulates the immune response by upregulating pattern recognition receptors (TLRs and NLRs), which, in turn, promotes robust T‐cell activation and the production of neutralizing antibodies, ultimately conferring strong cellular and humoral immunity in both mouse and cat models. **(C)** Microencapsulation of γ‐aminobutyric acid (GABA) and *Lactobacillus plantarum* (LP) by spray drying. (a) Experimental setup, materials used for co‐encapsulation, and optical micrographs of the resulting microcapsules with an optimized wall formulation. (b) Viability of LP and retention of GABA within the optimized microcapsules during storage at 4°C. EPS, exopolysaccharide. *Source*: (A) Reprinted from Gupta et al. (2024) with permission from Elsevier Ltd., (B) Reproduced from S. Zhang, Fan, et al. (2024) with permission from American Chemical Society, and (C) figure reprinted from Pandey and Mishra (2021) with permission from Elsevier Ltd.

An exemplary case of targeted delivery is the conjugation of curcumin to *Bacillus megaterium* EPS through succinic acid linkers (Gupta et al. 2024). The micelles demonstrate a significant pH‐dependent release profile, with 87.5% of their cargo released at acidic pH, characteristic of tumor microenvironments or lysosomes, in contrast to neutral pH (Figure 8A). The responsiveness observed is reflected in quercetin‐loaded nanoparticles sourced from *Pseudomonas otitidis*, which employ cross‐linking density to regulate release rates (Kalimuthu et al. 2023). These systems convert EPS from basic carriers into “intelligent” delivery vehicles that enhance therapeutic bioavailability and reduce systemic loss.

Furthermore, the structural modification of EPS can reveal inherent immunomodulatory functions in these delivery systems. Sulfated EPS nanoparticles derived from *Lactobacillus* demonstrate significant adjuvant properties, facilitating the transport of antigenic proteins while also actively engaging PRRs on macrophages (Figure 8B) (S. Zhang, Fan, et al. 2024). The dual role of functioning as both a carrier and an immunostimulant is essential for vaccine development, as demonstrated by the elicitation of strong cellular and humoral immunity against SARS‐CoV‐2 in animal models.

Finally, the protective capacity of EPS matrices encompasses the co‐encapsulation of live probiotics and various bioactives. Thermostable matrices consisting of dextran and inulin have been developed to safeguard both *L. plantarum* and GABA during the spray‐drying process (Figure 8C) (Pandey and Mishra 2021). The preservation of viability and bioactive content over several months of storage indicates that EPS matrices create a stabilizing microenvironment, effectively protecting sensitive components from oxidative and thermal stress. These applications highlight the evolution of microbial EPS from basic texturizers to advanced functional nanomaterials that facilitate innovation in drug delivery and food security.

## 6 Applications of Microbial EPS in Food

EPS serves an essential techno‐functional purpose in food systems, largely attributed to their hydrocolloidal characteristics and significant ability to bind water and retain moisture. Furthermore, the growing recognition of the health‐related advantages of specific prebiotic candidates drives the development of value‐added, functional foods. The functionalities are inherently associated with the molecular characteristics of the polysaccharide, such as its composition, molecular mass, chain conformation, and the presence and density of branching. Intrinsic viscosity ([*η*]) serves as a critical parameter indicative of these molecular attributes (Bai et al. 2017). This value measures the hydrodynamic volume occupied by a polymer in solution and indicates a polysaccharide's thickening capacity. High intrinsic viscosity generally indicates the presence of larger molecules with rigid chain structures, whereas lower values are associated with smaller, more flexible polymer chains.

EPS has a wide range of applications in multiple food sectors, particularly in the dairy and cereal industries, as well as in the emerging market of nondairy functional beverages. Furthermore, the growing recognition of their health‐related advantages drives the development of value‐added, functional foods. This trend corresponds with contemporary consumer preferences for natural, healthy alternatives that minimize additive content, establishing EPS as essential elements in the advancement of nutritious and innovative food solutions.

### 6.1 Dairy Products

Microbial EPS, especially those produced by LAB, are extensively utilized in the dairy industry to enhance textural characteristics, minimize syneresis, and improve the overall quality of various products (W. Liu, Wei, et al. 2025).

#### 6.1.1 Yogurts: Enhancing Texture, Viscosity, and Syneresis Control

LAB EPS plays a critical role in yogurt production by influencing texture, viscosity, and stability, thereby enhancing mouthfeel and prolonging shelf life (W. Liu, Wei, et al. 2025). These biopolymers serve as natural bio‐thickening agents, effectively stabilizing dairy ingredients while enhancing the viscosity and elasticity of the final product (Nikitina et al. 2023). Their robust interaction with positively charged casein, especially regarding negatively charged EPS, can yield a superior texture even at minimal concentrations. The complex interactions of various types of EPS influence yogurt's microstructural and rheological properties, facilitating advanced texture engineering and enabling targeted customization of characteristics such as firmness, creaminess, and syneresis resistance. This capability advances from general enhancement to focused product design that aligns with particular consumer preferences (Nikitina et al. 2023).

The research has examined the specific effects of different EPS‐producing strains on the network mechanics and syneresis of yogurt. For example, one study examined a negatively charged capsular EPS from *Streptococcus thermophilus* alongside a neutral, non‐capsular EPS from *Lactococcus lactis* (Brüls et al. 2024). The EPS from *S. thermophilus* improved casein interconnectivity and increased gel stiffness, whereas the EPS from *L. lactis* resulted in thicker protein domains, a higher number of small pores, and a reduced loss tangent. The combination of both strains resulted in a synergistic effect on microstructural and rheological properties. This finding provides important insights for the production of yogurts with customized microstructures, defined rheological properties, and improved resistance to syneresis. Subsequent studies on *S. thermophilus* strains (ST 285 and ST 1275) revealed differing effects of EPS on yogurt quality. Strain ST 1275, which produces a ropy‐capsular EPS, demonstrated enhanced growth and acidification rates, leading to yogurt characterized by reduced syneresis and an increased flow behavior index in comparison to yogurt produced with the capsular ST 285 strain (Purwandari et al. 2007). EPS production correlated with microbial growth, achieving maximum yields at designated fermentation temperatures (37°C for ST 285 and 42°C for ST 1275). Nevertheless, EPS concentration decreased markedly during extended cold storage, whereas cold storage enhanced various rheological properties, including storage modulus (*G*′), consistency index, and hysteresis loop area. A weak correlation between EPS concentration and textural properties indicates that the qualitative characteristics and specific structures of EPS are as important as their quantity in determining the final product attributes (Purwandari et al. 2007). This highlights that merely increasing EPS production is insufficient; the specific structural characteristics are crucial for attaining desired outcomes.

In addition to their functional roles in yogurt texture, specific EPS provide direct health benefits. A clinical study demonstrated the effectiveness of EPS‐enriched milk fermented by *Weissella confusa* VP30 (VP30‐EPS) in improving functional constipation (Jin et al. 2023). Participants who consumed VP30‐EPS exhibited an increase in both frequency and volume of defecation, as well as an enhancement in fecal type. Analysis of the gut microbiome revealed a transition to a more favorable microbial composition, marked by a modest increase in *Bacteroidetes* and *Prevotella* populations, alongside a slight reduction in Firmicutes. This clinical validation indicates that EPS‐enriched fermented milk can directly alleviate a specific gastrointestinal disorder, surpassing theoretical or in vitro benefits and offering substantial evidence for its therapeutic potential. The efficacy of functional ingredients in human trials represents a crucial advancement in product development, facilitating more robust health claims and enhancing consumer trust (Jin et al. 2023).

#### 6.1.2 Fermented Milk: Improving Rheology, Stability, and Consumer Acceptance

EPS produced by specific bacterial strains markedly improves the rheological characteristics and stability of fermented milks, which influences consumer acceptance via enhanced texture and flavor profiles. Fermented milks exhibit pseudoplastic (shear‐thinning) behavior, which is advantageous for processing and consumption. The production of EPS significantly affects this property; strains that produce higher quantities of EPS, especially high‐molecular‐mass (HMM) EPS, typically result in elevated apparent viscosities (De Vuyst et al. 2003). For example, *S. thermophilus* LY03 and CH101, *L. helveticus* LH18, and *L. mesenteroides* XR1, recognized for their high EPS yields, exhibited increased apparent viscosities (De Vuyst et al. 2003; L. Wang et al. 2023; K. Zhang, Liu, et al. 2024). The direct incorporation of purified EPS also contributes to the observed increase in viscosity. The molecular characteristics of EPS, including molecular mass, structure, and chain rigidity, serve as more significant determinants of rheological impact than the quantity of EPS produced. Some *S. thermophilus* strains demonstrate significantly different EPS yields while exhibiting comparable apparent viscosities (De Vuyst et al. 2003). In contrast, high‐Mw, rigid, and linear EPS, exemplified by XR1‐EPS (4.19 × 10^6^ Da with a rod‐like rigid chain structure), consistently results in increased viscosity, underscoring that the quality of EPS, rather than merely its quantity, determines the rheological properties (L. Wang et al. 2023).

Beyond rheology, EPS‐producing cultures enhance the quality of fermented milk products by improving water‐holding capacity (WHC) and decreasing syneresis. EPS accomplishes this by binding water through its hydroxyl groups and enhancing the protein gel network, resulting in a denser, more interconnected structure with smaller pores, as observed through scanning electron microscopy (K. Zhang, Liu, et al. 2024). Low‐field nuclear magnetic resonance demonstrates that EPS decreases the mobility of free water, resulting in enhanced water retention and stability. Furthermore, certain EPS strains, such as *L. mesenteroides* XR1, can mitigate post‐acidification during storage by competing for nutrients with other starter cultures (L. Wang et al. 2023). This results in reduced organic acid production, thereby extending shelf life and enhancing flavor.

The impact of EPS on consumer acceptance is substantial, as it notably affects the sensory texture of fermented milk. Products that include EPS typically demonstrate enhanced ropiness, smoothness, and a more defined cut, with a significant correlation identified between smoothness and ropiness (Hamet et al. 2015). The characteristic ropiness is a direct manifestation of the molecular properties of EPS and its interaction with the protein matrix, translating technological properties into consumer perception. Additionally, EPS can enhance the volatile flavor profile. *L. mesenteroides* XR1 enhanced the aroma of ketones and alkanes following 7 days of storage, thereby enriching the flavor profile of the yogurt (L. Wang et al. 2023). This indicates a comprehensive effect on sensory quality, likely resulting from metabolic changes or interactions that preserve or generate certain aroma compounds. The functional properties of EPS depend significantly on their molecular characteristics, such as quantity, molecular mass, sugar composition, molecular structure (including repeating units, branching, and linkages), and charge. High‐Mw, rigid, and linear chain structures typically result in increased viscosity and gel strength. The relationship between the structure and function of EPS is complex and influenced by multiple factors, requiring thorough molecular characterization for precise performance predictions, as mere quantity does not consistently align with functional outcomes (De Vuyst et al. 2003; L. Wang et al. 2023; K. Zhang, Liu, et al. 2024).

### 6.2 Nondairy Functional Beverages: Enhancing Quality With Microbial EPS

Microbial EPS are increasingly acknowledged as functional additives in nondairy beverages, providing numerous health benefits and enhancing product attributes (Bisson et al. 2025). Biopolymers produced naturally can improve the attractiveness of plant‐based fermented foods, especially for those with lactose intolerance or milk allergies.

Recent research emphasizes the potential of coconut water as a base for innovative functional beverages, capitalizing on their underutilized status. A study demonstrated the successful fermentation of coconut water using *L. plantarum* SVP2, with the addition of soybean protein and sucrose, resulting in a drink rich in EPS (Goveas et al. 2021). Under optimized conditions (27°C, 100 RPM for 36 h; 40 g/L sucrose, 0.75 g/L soybean protein, 1.5% v/v inoculum), a high EPS concentration of 64.17 ± 0.61 g/L was achieved. The fermented beverage exhibited substantial shelf stability, preserving cell count, pH, and EPS concentration for 7 days when refrigerated. Sensory analysis indicated moderate acceptance regarding flavor and texture; however, color and a slightly sour aroma were identified as areas needing improvement. The synergistic advantages of probiotic bacteria alongside the antibacterial and anti‐biofilm characteristics of EPS render this a promising functional food.

In addition to these findings, another study examined the effects of *Pediococcus acidilactici* EPS in a comparable fermented coconut beverage (Adebayo‐Tayo et al. 2024). This strain generated 6204.50 mg/L of EPS, identified as a flake‐like heteropolymer abundant in hydroxyl and carboxyl groups, with glucose as the predominant monosaccharide. This EPS demonstrated significant dose‐dependent antioxidant activity, as evidenced by DPPH radical scavenging, ferric ion‐reducing power, and total phenolic content assays. The *P. acidilactici* strain exhibited moderate tolerance to gastric conditions, including low pH, bile salts, and gastric juice, indicating its potential viability as a probiotic in the gastrointestinal tract. The high coaggregation capabilities suggest a potential for pathogen exclusion. The composition of the formulated beverage (pH, lactic acid, total soluble solids, and minerals) exhibited variation during storage; however, the incorporation of EPS significantly enhanced the protein content. The studies highlight the effectiveness of fermented coconut water as a medium for delivering beneficial probiotics and EPS, which possess various health‐promoting properties.

Research on a biofunctional fermented soy beverage, supplemented with microbial EPS produced by *L. mesenteroides*, demonstrated positive effects on the human gut microbiome in vitro (Bisson et al. 2025). The soy beverage fermented with *Lactiplantibacillus paraplantarum* GB3, supplemented with EPS (SMO or SMF), significantly increased the alpha diversity of the fecal microbiota following 24 h of incubation. SMO specifically enhanced the proliferation of beneficial species, including *Bifidobacterium longum* and *F. prausnitzii*, the latter recognized as a next‐generation probiotic. Fecal samples fermented with EPS‐supplemented beverages exhibited significantly higher levels of butyrate and propionate compared to the non‐supplemented control, indicating notable prebiotic potential and modulation of the gut environment. The study suggests that EPS, when used as a functional food ingredient, can enhance the gut microbiome and aid in the production of beneficial SCFAs.

These studies collectively point out the merits of microbial EPS in the development of innovative nondairy functional beverages. EPS has the potential to enhance nutritional value, improve gut health through microbiome modulation, and increase beneficial metabolites, thereby providing therapeutic properties that position these beverages as significant contributors to the functional food market.

### 6.3 Bread: Impact on Loaf Volume, Shelf Life, and Staling

LAB‐produced EPS enhances bread quality by increasing loaf volume, prolonging shelf life, and minimizing staling (Lynch et al. 2018a). This advantage is due to their ability to bind water and interact with components of the dough. The specific EPS type and LAB strain are essential, as their structural properties and metabolic activities, including acid production, affect outcomes. Dextran, for example, enhances dough stability and gas retention by forming a polysaccharide network that interacts with gluten, resulting in increased specific volume (Lynch et al. 2018a). EPS can delay staling by binding water and retaining moisture, thereby retarding starch crystallization. Microscopic images reveal that EPS is closely associated with starch granules, creating a film‐like structure that may impede water loss.

A comparative analysis of bread and steamed bread revealed the functionality of EPS (Xu et al. 2020). Sourdoughs fermented with EPS‐producing *Fructilactobacillus sanfranciscensis*, *W. cibaria*, and *L. mesenteroides* exhibited enhancements in specific volume and texture while also demonstrating a reduction in staling across both bread types. The effect was more significant in steamed bread, probably because of the unrestricted expansion of dough during steaming as opposed to baking. *F. sanfranciscensis* significantly enhanced bread quality irrespective of EPS, likely attributable to modifications in gluten. Following a 5‐day storage period, bread produced with *F. sanfranciscensis* and EPS‐containing sourdoughs derived from *W. cibaria* or *L. mesenteroides* demonstrated reduced crumb hardness. Steamed breads exhibited a greater enthalpy change, which aligns with observed staling rates. This study offers significant insights for enhancing commercial bread production.

Further investigation into the quality of Chinese steamed bread (CSB) utilizing *W. confusa* QS813 indicated that sourdoughs containing sucrose fermented at 20°C (WS20+) exhibited the highest EPS content and the lowest titratable acidity (Tang et al. 2018). WS20+ enhanced dough proofing and facilitated the agglomeration of glutenin macropolymer particles. Microstructural analysis indicated a more continuous network in WS20+ dough, leading to an improved crumb structure in CSB. WS20+ improved the quality of CSB, whereas low EPS without sucrose had detrimental effects. Elevated acidification at 35°C obscured the advantages of EPS. Consequently, elevated EPS production and moderate acidification enhanced the quality of CSB.

EPS‐producing *W. cibaria* NC516.11 also significantly enhanced the quality of buckwheat bread (J. Zhang, Yao, et al. 2023). This strain resulted in a 26.03% increase in specific volume and a 19.94% reduction in baking loss relative to controls. Scanning electron micrographs revealed a more intact and less porous crumb structure, suggesting improved gas retention. Fermentation significantly enhanced bread texture by decreasing hardness and minimizing moisture loss during storage. The improvements target prevalent challenges associated with gluten‐free bread, prolonging shelf life and improving sensory characteristics (J. Zhang, Yao, et al. 2023).

## 7 Limitations and Future Perspectives

Although microbial EPS have recognized potential in food and health applications, substantial challenges hinder their industrial and clinical implementation. This section assesses existing limitations and suggests future research approaches to fully realize the potential of these versatile biopolymers.

### 7.1 Current Limitations and Research Gaps

Despite extensive research over several decades, the complete commercial and therapeutic potential of the vast structural diversity of microbial EPS remains largely unexploited. The field faces limitations due to a combination of economic, analytical, and clinical challenges that need to be resolved to promote innovation.

#### 7.1.1 Techno‐Economic Constraints on Industrial Scalability and Innovation

The primary obstacle to the commercialization of novel microbial EPS is economic in nature. Although certain polysaccharides, such as xanthan, gellan, and dextran, have attained industrial success, they are exceptions rather than the norm (Freitas et al. 2017). High production costs represent a significant barrier for most new EPS, influenced by various interconnected variables. Fermentation typically necessitates costly, refined substrates, and the subsequent downstream processing, which includes cell separation, precipitation, purification, and drying, is complex and energy‐intensive (Freitas et al. 2017; J. Kumari et al. 2025; L. Liu, Zhang, et al. 2025). Additionally, numerous microbial strains that generate distinctive or bioactive EPS demonstrate low volumetric productivity under standard conditions, making industrial scale‐up economically impractical and increasing per‐unit costs (L. Liu, Zhang, et al. 2025).

This economic condition establishes a self‐reinforcing cycle that inhibits innovation. High production costs make it difficult to produce the gram‐to‐kilogram quantities needed for characterization, functional testing, and human clinical trials (J. Kumari et al. 2025; L. Liu, Zhang, et al. 2025; J. Wu, Han, et al. 2023). Securing substantial investment to optimize production and reduce costs is nearly impossible without rigorous validation that demonstrates a distinct market advantage or health benefit. This paradoxical situation confines promising EPS to the laboratory scale, preventing the generation of necessary data for commercialization and thereby reinforcing the market dominance of established incumbents (Freitas et al. 2017; L. Liu, Zhang, et al. 2025).

#### 7.1.2 The Structure–Function Paradox, an Intricate Aspect of Biological Design

A critical knowledge gap regarding the structure and function of EPS constitutes a substantial scientific obstacle to innovation. The structural elucidation of these polymers is inherently challenging due to their complex characteristics, including high Mw, heterogeneity, and intricate branching patterns (L. Liu, Zhang, et al. 2025; Schmid et al. 2016). The complexity of EPS solutions obstructs standard analytical techniques. For example, their high viscosity frequently results in broadened spectral lines in NMR spectroscopy, which complicates the accurate identification of glycosidic linkages and repeating unit structures. This analytical constraint results in the rare attainment of complete, high‐resolution structures for novel EPS (Schmid et al. 2016).

The presence of analytical uncertainty hinders the advancement of predictive structure–function models. The correlation between particular structural characteristics and functional properties, including gelling ability and prebiotic activity, is predominantly based on empirical evidence (L. Liu, Zhang, et al. 2025; Schmid et al. 2016). The challenge is exacerbated by a concurrent deficiency in our comprehension of EPS biosynthesis. The characteristics of essential enzymes, such as GTs, remain largely unidentified, particularly concerning EPS derived from novel sources like fungi or archaea. A clear understanding of both the final structure and the biosynthetic pathway is essential for the rational design and targeted bioengineering of EPS with specific properties (Schmid et al. 2016).

#### 7.1.3 The Challenge of Translating Preclinical Discoveries Into Human Health

A significant gap exists between the abundance of promising preclinical data for microbial EPS and the lack of substantial human clinical validation (Cunningham, Vinderola, et al. 2021). Research has focused on established oligosaccharides, such as FOS and GOS, as well as whole probiotics; however, the clinical benefits of most isolated and purified EPS remain unsubstantiated (Cunningham, Vinderola, et al. 2021).

This translational gap is exacerbated by a significant mechanistic ambiguity. The advantages of EPS are primarily linked to their prebiotic role, facilitating selective fermentation by gut microbiota to generate beneficial SCFAs. The emerging evidence, however, indicates that polymers like fungal β‐glucans may interact directly with gut mucosal receptors, independent of fermentation processes (Araújo‐Rodrigues et al. 2024; Ayimbila and Keawsompong 2025). It is essential to accurately apply the official ISAPP definition, as “postbiotics” are frequently conflated with other terms. A postbiotic is defined as a “preparation of inanimate microorganisms and/or their components that confers a health benefit on the host” (Salminen et al. 2021). Highly purified EPS, once isolated from cellular biomass, are more appropriately termed “bioactive biopolymers” or “immunomodulators” instead of postbiotics, despite potential similarities in downstream signaling mechanisms. This distinction is essential for regulatory clarity and claims regarding the mechanism of action.

Current clinical trials are insufficiently designed to elucidate prebiotic and postbiotic pathways. Observing reduced systemic inflammation following EPS administration raises a critical question: Is this driven by downstream SCFA production (prebiotic) or direct immunomodulation by the polymer (bioactive)? This uncertainty undermines the scientific basis for health claims and poses a significant barrier to regulatory approval from organizations such as the European Food Safety Authority (EFSA) or FDA. Addressing this necessitates advanced trial designs that longitudinally assess the polymer's fate, microbial metabolic changes, and particular host immune responses. The lack of such evidence significantly limits the capacity to promote EPS as high‐value functional food ingredients (Araújo‐Rodrigues et al. 2024; Ayimbila and Keawsompong 2025; Cunningham, Vinderola, et al. 2021; J. Wu, Han, et al. 2023).

#### 7.1.4 Inherent Deficiencies of Native Polymers in Industrial Settings

A practical limitation is that many native microbial EPS, despite their potential health benefits, have intrinsic physicochemical properties that are not optimal for industrial food processing. Native polymers often demonstrate inadequate solubility in aqueous environments, reduced thermal stability, increased vulnerability to degradation under mechanical shear, and a tendency for microbial contamination, which restricts their application as food ingredients (Ahuja et al. 2023). To address these deficiencies, native EPS typically necessitates chemical modification (e.g., acetylation and sulfation) or must be incorporated into composites with other natural or synthetic polymers to improve properties such as mechanical strength or stability (Ahuja et al. 2023; L. Liu, Zhang, et al. 2025). Although these strategies may prove effective, they also entail additional processing steps, elevate overall costs, and potentially generate regulatory challenges. Moreover, these modifications detract from the “natural” and “clean label” status that significantly influences consumer interest in microbial biopolymers, consequently undermining one of their main market advantages (Ahuja et al. 2023).

#### 7.1.5 Regulatory Landscapes for Microbial EPS

The commercial translation of microbial EPS from laboratory potential to functional ingredient depends on navigating various regulatory frameworks, particularly the European Union (EU) Novel Food regulation and the United States (US) generally recognized as safe (GRAS) and new dietary ingredient (NDI) pathways. For product developers, comprehending these distinct mechanisms is a crucial techno‐economic factor for market entry.

In the EU, EPS without a substantial consumption history before May 1997 are categorized as novel foods according to Regulation (EU) 2015/2283 (Le Bloch et al. 2025). Authorization necessitates a centralized safety assessment by the EFSA, requiring detailed data on the polymer's identity, production process, and toxicological profile (Nutrition et al. 2021). This pathway is rigorous and significantly time‐consuming; recent analyses show that the average time from submission to an EFSA scientific opinion is around 2.56 years (Le Bloch et al. 2025). The qualified presumption of safety (QPS) status of a producing microorganism may facilitate the safety assessment of the biomass; however, the isolated EPS structure generally requires a comprehensive novel food assessment (Allende et al. 2025).

In contrast, the US employs a dual, risk‐based system that varies according to the intended use of the product. The EPS must be established as GRAS for conventional foods (Food and Drug Administration 2016). This designation necessitates “general recognition” of safety by qualified experts, grounded in publicly accessible scientific evidence. Developers may opt for self‐affirm GRAS to facilitate rapid market entry or submit a voluntary notification to the FDA to achieve enhanced regulatory certainty (National Institutes of Health 1994). Developers may alternatively utilize the NDI pathway established by DSHEA for exclusive use in dietary supplements (National Institutes of Health 1994). This required notification, submitted 75 days before marketing, permits the use of confidential, proprietary data to establish that the ingredient is reasonably anticipated to be safe (Food and Drug Administration 2024).

These differences frequently lead to a “US‐first” commercialization strategy. The capacity to initiate operations in the US through Self‐GRAS or NDI notification enables companies to generate revenue and collect market data while concurrently seeking the multi‐year authorization necessary for the EU. Successful approval, irrespective of jurisdiction, depends on consistent manufacturing quality and thorough characterization of the producing strain.

### 7.2 Future Perspectives: Charting the Path Forward

Addressing the complex challenges mentioned necessitates a collaborative, interdisciplinary research approach. The future direction entails utilizing microbial biodiversity, adopting advanced engineering and analytical methodologies, ensuring thorough clinical validation, and formulating novel strategies for food applications.

#### 7.2.1 Utilizing Microbial Diversity and Engineering for “Designer” Polysaccharides

The future of EPS development is characterized by a significant shift from the passive discovery of natural polymers to the deliberate design of polysaccharides with tailored functionalities. This objective can be achieved through various complementary strategies.

A promising area of research involves systematic bioprospecting of unexplored microbial ecosystems, especially in extreme environments such as deep‐sea hydrothermal vents, hypersaline lakes, and polar ice. These efforts can produce novel EPS characterized by unique structures and robust properties, including thermostability and salt tolerance, which are advantageous for challenging industrial processing conditions. Concurrently, to ensure economic viability, production must shift from costly, pure substrates to low‐cost, sustainable feedstocks. This entails the engineering of resilient microbial chassis, such as *E. coli* or *B. subtilis*, to effectively valorize agro‐industrial waste streams, including lignocellulosic biomass, whey permeates, or crude glycerol, thus situating EPS manufacturing within a sustainable circular bioeconomy (Aggarwal et al. 2023).

The ultimate strategy involves utilizing synthetic biology to develop entirely new biosynthetic pathways. The development and validation of a modular toolkit comprising genes that encode enzymes with established specificities enable precise control over the primary structure of an EPS, encompassing its monomer sequence, linkage types, branching patterns, and Mw (Becker 2015). This approach facilitates the systematic synthesis of polymer libraries by varying single parameters, thereby offering a robust, hypothesis‐driven methodology to elucidate the complex relationship between polysaccharide architecture and its biological and physical properties.

#### 7.2.2 Integrating Advanced Analytical and Predictive Tools to Decode Complexity

Advancements in the engineering of new polysaccharides should coincide with improvements in characterization techniques. Future research should utilize a systems‐level approach that integrates multi‐omics data, including genomics, transcriptomics, proteomics, and metabolomics, alongside advanced analytical techniques. This involves the application of advanced high‐field NMR spectroscopy alongside sophisticated molecular modeling and simulation to elucidate complex structures from complicated spectra (Schmid et al. 2016). Moreover, advances in high‐resolution imaging techniques, such as cryo‐electron microscopy (cryo‐EM), offer an exceptional perspective of the entire multi‐protein complex involved in EPS production and export within the cell membrane. This insight will provide a thorough understanding of the process from genes to final polymer, guiding future engineering strategies.

In parallel, the development and application of machine learning (ML) and artificial intelligence (AI) can significantly expedite the research and development (R&D) pipeline. Training algorithms on the expanding dataset of genomic, structural, and functional information enables the development of ML models that can predict the structure and potential bioactivity of an EPS directly from a microbe's genome sequence (Becker 2015; W. Liu, Wei, et al. 2025). These in silico models can also identify the most promising candidate strains for laboratory screening and recommend optimal fermentation conditions to enhance yield.

#### 7.2.3 A Strategic Roadmap for Clinical Validation and Regulatory Success

A comprehensive strategic roadmap is essential to connect the preclinical potential of microbial EPS with market realities. This strategy necessitates the establishment and widespread acceptance of standardized protocols for EPS production, characterization, and bioactivity testing, in conjunction with the implementation of advanced preclinical models such as Gut‐on‐a‐Chip platforms to more accurately replicate human gut physiology (Kim and Sung 2024). The emphasis should transition to conducting rigorous, placebo‐controlled human trials that extend beyond basic feasibility to evaluate clinically significant endpoints in metabolic, immune, and gastrointestinal health while also investigating innovative targets like the microbiota‐gut–brain axis.

A significant paradigm shift involves transitioning from a “one‐size‐fits‐all” approach to personalized interventions, wherein an individual's microbiome profile informs the choice of a specific EPS to attain a targeted functional outcome (Gibbons et al. 2022). Clinical trials should prioritize mechanistic interrogation as a fundamental objective, employing multi‐omics approaches to establish a system‐level perspective that links microbial shifts to clinical outcomes, thus elucidating prebiotic and postbiotic effects. This robust, mechanism‐based evidence is essential for substantiating health claims, navigating the intricate regulatory challenges to obtain approvals such as GRAS status, and effectively positioning next‐generation functional ingredients within the commercial market.

#### 7.2.4 Clinical Perspective and Future Developments

The current state of microbial EPS research is marked by an apparent paradox: Preclinical data consistently indicate significant health benefits, yet there is a lack of substantial human clinical validation, as research has traditionally focused on established plant‐derived oligosaccharides or whole probiotics (Cunningham, Azcarate‐Peril et al. 2021). Currently, a significant paradigm shift is occurring as these biopolymers—previously appreciated mainly for their rheological properties—are being reassessed as effective physiological modulators. The advancements are particularly notable in the research on yeast β‐glucans, where substantial clinical evidence now substantiates their involvement in immune training and metabolic regulation (De Marco Castro et al. 2021).

Similarly, bacterial EPS are increasingly recognized for their clinical applications beyond their conventional functions. Levan has emerged as a leading candidate, with human trials confirming its safety and demonstrating its bifidogenic potential and capacity to enhance bowel regularity (Alshammari et al. 2024; Ostrowski et al. 2022). Moreover, the “technological” gums xanthan and gellan, once considered non‐fermentable, are now acknowledged in clinical contexts as active substrates that serve as primary nutrition for certain gut commensals, thereby initiating cross‐feeding networks that generate beneficial SCFAs (Alshammari et al. 2024; Ostrowski et al. 2022). This reclassification signifies a substantial extension of the prebiotic repertoire from basic oligosaccharides to intricate, high‐Mw polymers.

Despite these developments, technical constraints and mechanistic uncertainty still hinder the conversion of EPS into medicinal uses. Although prebiotic fermentation is often associated with advantages, new research indicates that polymers such as fungal β‐glucans may function via direct signaling pathways, independent of fermentation. This activity corresponds with the concept of signaling by nonviable microbial components; however, it is more accurate to classify purified EPS as “bioactive biopolymers” rather than postbiotics, which generally refers to preparations of inanimate cells. Clear mechanistic evidence is necessary to differentiate this direct activity from prebiotic effects (Rastall and Gibson 2015). Current studies often suffer from structural ambiguity in commercial preparations and are unable to differentiate between these routes (Rastall and Gibson 2015). Furthermore, since prebiotic efficiency depends on the host having certain primary degraders (such as *Ruminococcaceae* for xanthan gum) and because highly effective dosages might impair acceptability, the “one size fits all” strategy typically produces “nonresponder” phenotypes (Niv et al. 2012; Ostrowski et al. 2022).

Future research should focus on precision and standardization to address this translational gap. Advanced trial designs ought to integrate “responder analysis” for the stratification of participants according to baseline microbiome biomarkers, facilitating personalized interventions instead of generic supplementation. Strategies should advance towards “synbiotics 2.0,” which involves the systematic pairing of specific EPS with established degrading strains to guarantee fermentation independent of the host's native microbiota (Cunningham, Azcarate‐Peril, et al. 2021; Miyamoto et al. 2023). Additionally, the standardization of production protocols and the integration of a multi‐omics approach to delineate fermentation pathways will be essential for validating health claims and addressing regulatory challenges.

#### 7.2.5 Innovative Food Applications: Integrating Microbial EPS Into the Modern Diet

**FIGURE 9:**
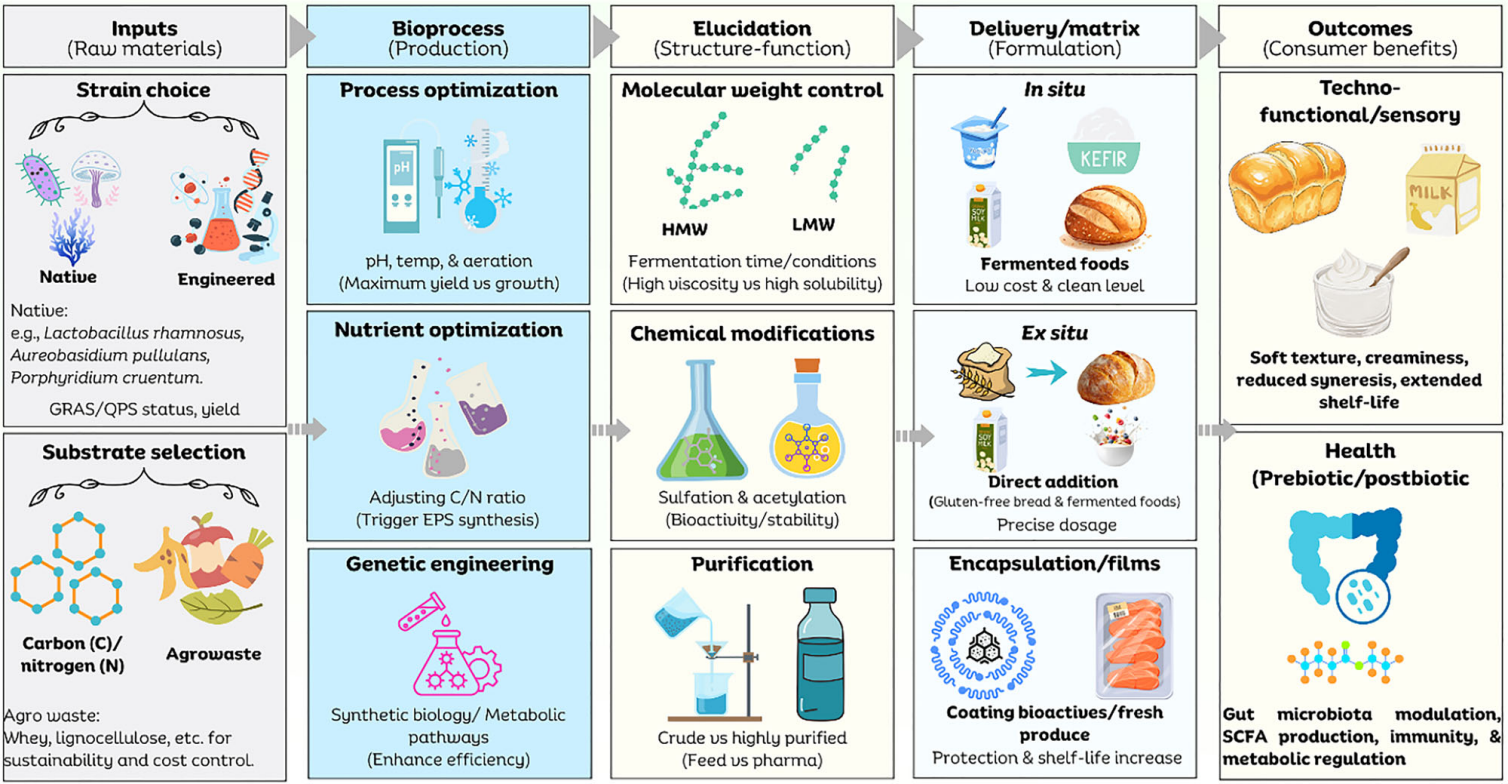
A strategic value‐chain paradigm for the development of functional foods made from microbial EPS. Figure shows the hierarchical flow from raw material selection to consumer benefits to help practitioners customize EPS functionality. To maximize techno‐functional qualities (texture and stability) and health outcomes (prebiotic/postbiotic effects), the procedure makes a distinction between in situ production (fermentation) and ex situ application (additives/packaging). EPS, exopolysaccharide. *Source*: Figure was created in Canva.com.

The effective integration of microbial EPS into the food supply relies on strategies that adeptly utilize their combined techno‐functional and health‐enhancing characteristics. Product developers have various approaches available, each tailored to specific food categories and value propositions. The decision to produce EPS in situ during fermentation, incorporate them as purified functional ingredients, or utilize them in advanced delivery systems provides significant flexibility in developing innovative, value‐added food products that align with contemporary consumer preferences for health, naturalness, and sustainability (Korcz and Varga 2021; Lynch et al. 2018b; J. Wu, Han, et al. 2023). Table 9 and Figure 9 present a comparative framework for strategic approaches that guide food product innovation.

**TABLE 9:** Strategic approaches for the incorporation of microbial exopolysaccharides (EPS) into food systems.

Application strategy	Mechanism of incorporation	Exemplary food products	Key benefits (techno‐functional and health)	References
**In situ production**	EPS are synthesized directly within the food matrix by probiotic or starter cultures during fermentation	Yogurt, kefir, fermented milks, certain cheeses, sourdough bread, fermented plant‐based alternatives (e.g., soy/oat yogurt)	**Techno‐functional**: Natural thickening, improved viscosity and mouthfeel, enhanced water‐holding capacity, reduced syneresis, “clean label” (replaces added hydrocolloids) **Health**: Delivers a synbiotic combination of live probiotics and candidate EPS; potential for enhanced microbial viability during gut transit	Brüls et al. (2024), Jin et al. (2023), Yu et al. (2021)
**Purified functional ingredient**	Extracted, purified, and standardized EPS are incorporated into food formulations as a bioactive powder	Low‐fat dairy and meat products (fat mimetic), gluten‐free bakery (structure enhancer), beverages (improves body/mouthfeel), sauces, dressings, and soups (stabilizer)	**Techno‐functional**: Precise control over dosage and functionality, standardized product quality, applicable to non‐fermented foods **Health**: Delivers a defined dose of a specific prebiotic; allows for targeted health claims based on the properties of the purified EPS	Bisson et al. (2025), Lynch et al. (2018a), Tang et al. (2018), L. Wang et al. (2023), J. Zhang, Yao, et al. (2023), K. Zhang, Liu, et al. (2024)
**Advanced delivery and packaging systems**	EPS serves as a fundamental material for encapsulation and as a constituent in active or edible packaging	Microencapsulation of probiotics, vitamins, and omega‐3 fatty acids; development of edible films and coatings applicable to fresh produce, meats, and cheeses	**Techno‐functional**: Protects sensitive bioactives from degradation, extends food shelf life by forming a barrier against moisture and gas, and minimizes plastic waste **Health**: Improves the bioavailability of encapsulated nutrients; facilitates the direct delivery of antimicrobial/antioxidant compounds to the food surface; offers prebiotic benefits upon consumption	Guerrero et al. (2024), Pandey and Mishra (2021), D. Zhao, Yu, et al. (2025)

## 8 Conclusion

Microbial EPS are a significant and versatile category of biopolymers, situated at the intersection of food technology and human nutritional science. This review systematically delineates their dual identity: They function as high‐performance techno‐functional ingredients that modify rheology, enhance texture, and stabilize complex food matrices, whereas specific candidate EPS serve as potent bioactive agents. Among these, EPS that meet the criteria of resistance and selective fermentation act as prebiotics, promoting beneficial shifts in the gut microbiota‐host axis and resulting in enhanced local and systemic health.

The increasing consumer demand for natural, clean‐label, minimally processed, and functional foods highlights the relevance of microbial EPS as a viable solution. Their capacity for production from renewable resources, particularly through the valorization of agro‐industrial waste streams, is consistent with the principles of a circular bioeconomy. This offers a sustainable and environmentally friendly alternative to petroleum‐based synthetic polymers and conventional plant‐derived hydrocolloids, whose production may compete for valuable agricultural land and resources.

However, the considerable potential of these biopolymers is hindered by substantial economic, analytical, and clinical challenges that restrict their wider application. Addressing these challenges requires the collaboration of multiple scientific disciplines. A concerted and integrated interdisciplinary effort is required, which involves the expertise of microbiologists, genetic engineers, analytical chemists, food scientists, clinical nutritionists, and bioprocess engineers. To fully realize the potential of microbial EPS, the scientific community must foster collaboration on rational design, sustainable production, and rigorous clinical validation. This collaborative effort will play a crucial role in developing healthier, more sustainable, and more functional foods that effectively address the requirements of an expanding global population.

## Author Contributions

**Md. Abdur Razzak**: conceptualization, methodology, software, data curation, investigation, visualization, writing – review and editing, writing – original draft. **Hye Kim**: data curation, formal analysis, validation, investigation, writing – original draft, visualization. **Md. Ariful Haque**: data curation, formal analysis, validation, investigation, writing – original draft. **Min Ji Jang**: data curation, formal analysis, validation, investigation, writing – original draft. **Sehyeon Song**: data curation, formal analysis, validation, investigation, writing – original draft. **Anishka Talari**: formal analysis, investigation. **Lakshmi Devi Chittepu**: investigation, formal analysis. **Seockmo Ku**: conceptualization, methodology, supervision, resources, project administration, formal analysis, validation, investigation, funding acquisition, writing – review and editing.

## Conflicts of Interest

The authors declare no conflicts of interest.
